# Dimensionally Stable Anion Exchange Membranes Based on Macromolecular-Cross-Linked Poly(arylene piperidinium) for Water Electrolysis

**DOI:** 10.1021/acsami.3c13801

**Published:** 2024-01-04

**Authors:** Xiuqin Wang, Angela Mary Thomas, Rob G. H. Lammertink

**Affiliations:** † Soft Matter, Fluidics and Interfaces, Faculty of Science and Technology, MESA+ Institute for Nanotechnology, University of Twente, 7522 NB Enschede, The Netherlands; ‡ School of Environment and Civil Engineering, Dongguan University of Technology, Dongguan 523808, P. R. China; § TECNALIA, Basque Research and Technology Alliance (BRTA), Mikeletegi Pasealekua 2, 20009 Donostia-San Sebastian, Spain

**Keywords:** macromolecular cross-linker, molecular dynamics simulation, limited swelling, microphase separation, water electrolysis

## Abstract

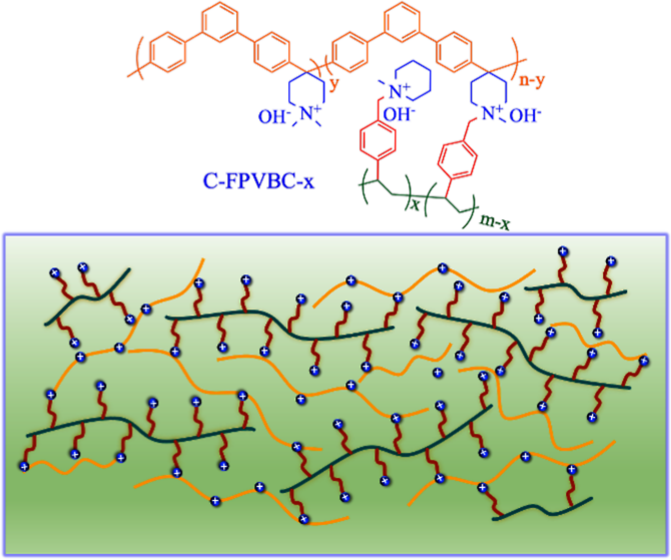

The advancement of anion exchange membranes (AEMs) with superior ionic conductivity has been greatly hindered due to the inherent “trade-off” between membrane swelling and ionic conductivity. To resolve this dilemma, macromolecular covalently cross-linked C-FPVBC-*x* AEMs were fabricated by combining partially functionalized ether-bond-free polystyrene (FPVBC) with poly(arylene piperidinium). The results from atomic force microscopy reveal that an increase in the ratio of FPVBC promotes the fabrication of microphase separation morphology, resulting in a high ionic conductivity of 40.15 mS cm^–1^ (30 °C) for the C-FPVBC-1.7 membrane. Molecular dynamics simulations further examine the ionic conduction effect of cross-linked AEMs. Besides, the unique cross-linking structure significantly improves mechanical and alkaline stability. After treatment in 1 M KOH at 50 °C for 1200 h, the C-FPVBC-1.7 membrane shows only a 6.9% decrease in conductivity. The C-FPVBC-1.7 AEM-based water electrolyzer achieves a high current density of 890 mA cm^–2^ at 2.4 V (80 °C) and maintains good stability, enduring over 100 h at 100 mA cm^–2^ (50 °C). These results demonstrate the significant potential of macromolecularly cross-linked AEMs for practical applications in water electrolysis.

## 1 Introduction

Addressing the pressing global energy crisis and environmental concerns stemming from human activities requires the development of sustainable and environmentally friendly energy conversion and storage technologies. To this end, ongoing efforts are directed toward the development of diverse energy conversion technologies, encompassing water electrolysis, lithium-ion batteries, fuel cells, etc.^1^ Among them, water electrolysis stands out as a promising energy conversion method, demonstrating promise in utilizing intermittent clean energy sources (e.g., solar energy, tides, and wind) to generate H_2_ from water.^2^ However, conventional hydrogen production from natural gas, coal, and biomass emits substantial greenhouse gases, such as CO_2_. To mitigate these emissions, more sustainable methods like water electrolysis can reduce costs and decrease the carbon footprint of hydrogen production.^3^

Anion exchange membrane water electrolysis (AEMWE) attracts great attention due to its accelerated kinetics for the O_2_ reduction reaction and the possibility of employing nonprecious metals (e.g., nickel) as electrocatalysts, leading to cost-effective hydrogen production.^4^ As a vital element in AEMWE, anion exchange membranes (AEMs) can separate O_2_ and H_2_ and transport OH^–^. To operate effectively at desired conditions, AEMs require improved hydroxide conductivity and excellent chemical stability in highly alkaline environments. They should also maintain adequate water uptake (WU) without dimensional changes to facilitate ion conduction.^5^ Thus, further research study is necessary to optimize the performance of AEMs in AEMWE technology for commercial hydrogen production. The conductivity of the AEMs falls behind that of proton exchange membranes due to the lower mobility of OH^–^. Increasing the ionic exchange capacity (IEC) of the AEMs has the potential to improve conductivity. Nonetheless, this often results in increased membrane swelling, compromising the mechanical properties. The challenge of balancing the ionic conductivity with membrane swelling remains unresolved.

Traditional AEMs often degrade in alkaline conditions due to the OH^–^ nucleophilic attack, which hinders their commercialization. Polyelectrolytic AEMs at the forefront of development use various polymers, including poly(arylene ether),^6^ poly(phenylene oxide),^7^ poly(olefin),^8−10^ poly(phenylene piperidinium),^11,12^ etc. In numerous instances, the durability of AEMs is impacted by the structures of both polymer backbones and cations.^13^ Cations are prone to degradation in alkaline environments, involving processes such as nucleophilic substitution, Hofmann degradation, and ylide degradation.^14^ Consequently, a decline in ionic conductivity/IEC is observed following treatment of the AEM with an alkaline solution. N-Heterocyclic ammonium functional groups could maintain stability in harsh environments, as identified by Lee’s group.^15^ Our previous work has examined how N-cyclic cations affect membrane performance.^16^ The results showed that less than 6% of IEC of piperidinium-functionalized AEMs decreased after being treated with 5 M NaOH (80 °C, 240 h). These results offer guidance for the development of alkaline-stable AEMs.

Besides the cation degradation, aryl ether-containing polyaromatics also exhibit a significant likelihood of experiencing backbone cleavage through exposure to an alkaline environment.^17^ This effect becomes more pronounced when electron-withdrawing groups are introduced into the neighboring phenyl ring, especially for the benzyl-based backbone with sulfone groups, which induces electron deficiency in the benzyl ring, thereby increasing susceptibility to backbone cleavage.^18^ To enhance the long-term stability of AEMs in AEMWE, one of the most promising approaches is to design AEMs with backbones devoid of ether bonds. The group of Yan designed a variety of poly(arylene piperidinium) AEMs with backbones free of ether bonds, showcasing outstanding chemical stability.^19^ The chemical structure of the AEM rarely changed after being treated with 1 M KOH (2000 h and 100 °C). Nevertheless, rigid backbones may render AEMs susceptible to brittleness and result in poor mechanical properties. Cross-linking is an effective approach to restrain membrane swelling and enhance mechanical performance. Previously, we employed a multication cross-linking agent to fabricate cross-linked AEMs.^20^ The resulting membranes demonstrated superior dimensional stability and conductivity compared to their non-cross-linked counterparts. Macromolecular cross-linkers possess more reactive functional groups compared to small-molecule cross-linkers, leading to a more compact cross-linking network structure.^21^ The group of Yi fabricated a variety of cross-linked AEMs using a macromolecular cross-linker based on poly(vinyl acetal) that contains ether bonds.^22^ However, a significant conductivity decline of almost 50% was observed when these AEMs were immersed in 1 M KOH at 40 °C. This susceptibility to degradation under alkaline conditions is attributed to ether bonds in AEMs.^17^ Thus, developing a novel macromolecular cross-linker with backbones free of ether bonds is highly desirable.

Motivated by the benefits of a backbone free of ether bonds and a macromolecular cross-linker, we employed poly(arylene piperidine) (PAP) as an ether-bond-free polymer and partially functionalized polystyrene (FPVBC) as the macromolecular cross-linker to prepare innovative cross-linked AEMs. FPVBC contains −CH_2_Cl groups in its repeating units that can react with the piperidine group, forming a cross-linking structure. By employing the cross-linking approach, we significantly alleviated the “trade-off” dilemma between membrane swelling and ionic conductivity. Molecular dynamics (MD) simulations were conducted to examine the ionic conduction effect of cross-linked AEMs. The AEMWE durability and performance were also explored in a C-FPVBC-1.7 AEM-based electrolyzer.

## 2 Experimental Section

### 2.1 Materials

1-Methylpiperidine (99%), CH_3_I (99%), *m*-terphenyl (99%), trifluoromethanesulfonic acid (98%), *N*,*N*-diisopropylethylamine (DIPEA, 99%), *N*-methyl-4-piperidone (97%), and poly(vinyl benzyl chloride) (PVBC) were purchased from Sigma-Aldrich. K_2_CO_3_ (99%), NaCl (99.5%), KOH (99.5%), NaNO_3_ (99%), AgNO_3_ (99%), dichloromethane (DCM, 99%), and dimethyl sulfoxide (DMSO, 99.8%) were obtained from VWR Chemicals. Ultrapure water was used throughout the experiments.

### 2.2 Membrane Preparation

#### 2.2.1 Synthesis of PAP

**Scheme 1:**
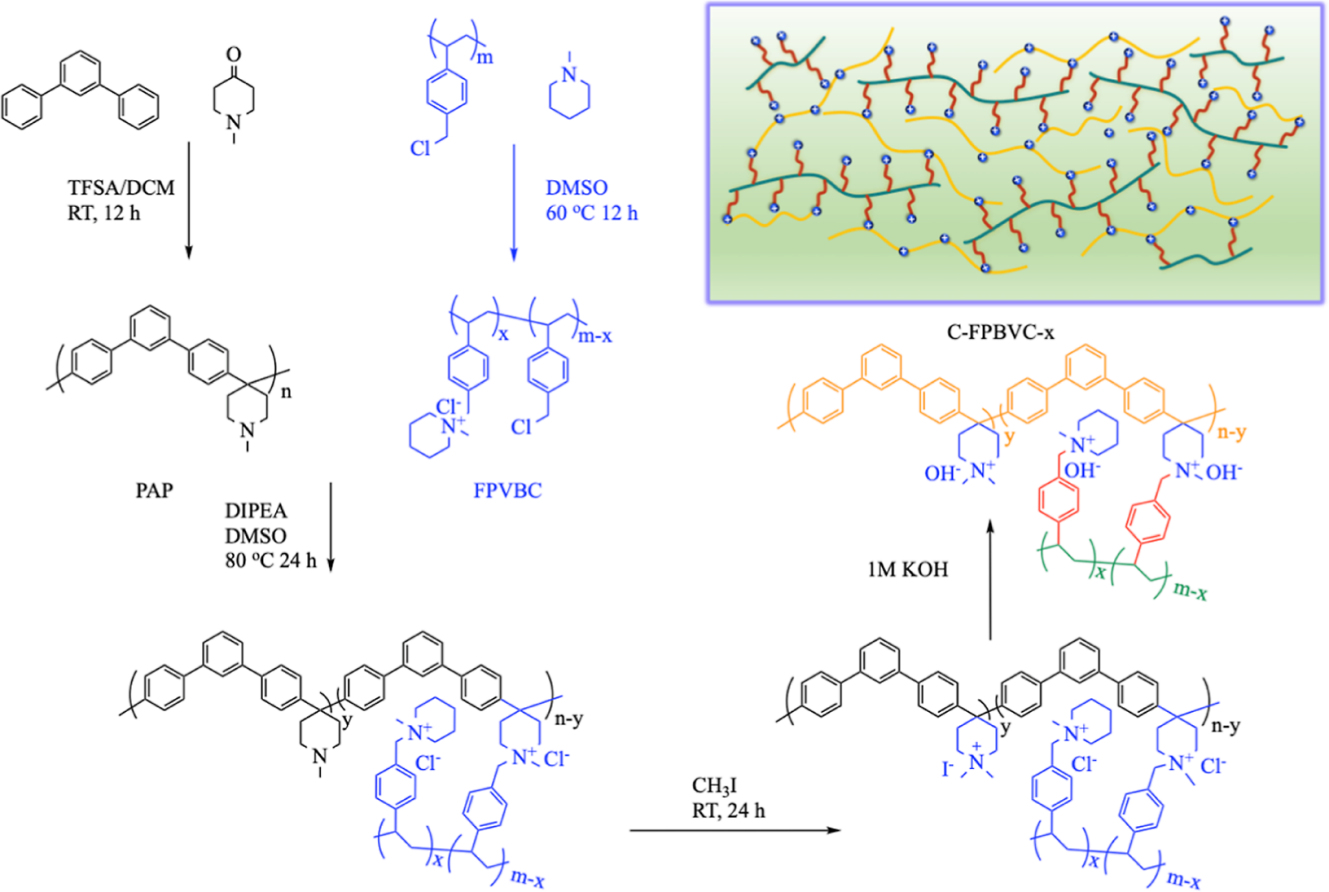
Synthetic Process for C-FPVBC-*x* Membranes

The synthetic process for PAP is illustrated in Scheme 1. In a flask equipped with an ice bath, 1.47 g of *N*-methyl-4-piperidone and 2.30 g of *m*-terphenyl were dissolved in 20 mL of DCM to form a mixture. Next, 8.80 mL of trifluoromethanesulfonic acid was added to the above mixture. The resulting brown solution turned viscous after 24 h, which was then precipitated in water. The resulting PAP was washed with water and dried at 60 °C.

#### 2.2.2 Synthesis of FPVBC

FPVBC with an 80% functionalization degree was synthesized following the method described by Lin et al.^9^ 1.83 g of PVBC and 0.80 g of 1-methyl piperidine were dissolved in DMSO to form a mixture, which was stirred at 60 °C for 24 h, resulting in the formation of a homogeneous FPVBC solution.

#### 2.2.3 Preparation of C-FPVBC-*x* AEMs

Membranes denoted as C-FPVBC-*x* (with *x* representing the molar ratio of −CH_2_Cl in FPVBC to the piperidine group in PAP, *x* = 0.7, 0.8, 1.0, 1.2, and 1.7) were produced. Using C-FPVBC-1.0 as a representative case, 0.14 g of PAP was placed in DMSO (4.0 mL) to fabricate the PAP solution. The FPVBC solution (1.0 mL), DIPEA (20 μL), and the PAP solution were mixed, then cast onto a glass plate, and dried at 80 °C for 48 h to yield a flexible membrane. The resulting membranes were soaked in a CH_3_I/ethanol mixture within a light-shielded condition at room temperature (RT) for 24 h. Subsequently, they were rinsed with water and treated with 1 M KOH for 48 h. The membranes were rinsed with water and stored in a vessel blanketed with a flowing N_2_.

### 2.3 Characterization and Measurements

^1^H NMR spectra were recorded on a 400 MHz Bruker ASCEND spectrometer. Fourier-transformed infrared (FT-IR) spectroscopy was conducted by using a PerkinElmer UATR spectrometer with a scanning range of 400–4000 cm^–1^. Tensile measurements were performed using a Zwick Z5.0 instrument with a crosshead speed of 5 mm min^–1^ at RT. Thermogravimetric analysis (TGA) was carried out using a TGA 4000 instrument (PerkinElmer) from 30 to 800 °C (10 °C min^–1^) under a N_2_ atmosphere. Scanning electron microscopy (SEM, JSM6010LA) and tapping-mode atomic force microscopy (AFM, Bruker Dimension Icon) were performed for examining the morphology of the membranes. Small-angle X-ray scattering (SAXS) experiments were conducted by employing a SAXS SYSTEM from XENOCS, France.

#### 2.3.1 Gel Fraction

The gel fraction (GF) was measured by soaking the samples in DMSO at 80 °C for 24 h. The residual samples were dried to a constant weight. The GF was obtained from the dry weights of samples before and after the immersion treatment.

#### 2.3.2 WU and Swelling Ratio (SR)

The membranes (OH^–^ type) were subjected to vacuum drying at 60 °C for 24 h to obtain the dry weight (*m*_dry_) and the dry length (*l*_dry_). Then, the membranes were soaked in water at a specific temperature to acquire the wet weight (*m*_wet_) and the length (*l*_wet_). Measurements were conducted on a minimum of three occasions to calculate average values. The WU_m_ (wt %) was calculated according to eq. 1
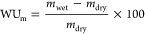
1

The volumetric WU_v_ (vol %) is calculated by eq. 2
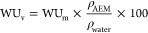
2ρ_AEM_ and ρ_water_ are the densities of the membrane and water, respectively.

The linear expansion (SR_l_) of the membranes is obtained from eq. 3
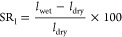
3

#### 2.3.3 IEC

The IEC_m_ of the AEMs is determined by utilizing the Mohr titration method. The mass of a dry membrane was measured, followed by immersing in a 2 M NaCl solution for 24 h to achieve a complete ion exchange with chloride ions. The membrane in the Cl^–^ form was then rinsed with ultrapure water and kept in 2 M NaNO_3_ (100 mL) for 24 h. Then, the content of Cl^–^ in the solution was measured by titrating the AgNO_3_ solution employing a Ag electrode in 805 Dosimat-Metrohm AG.

The titrated IEC_m_ (mequiv g^–1^) is calculated using eq. 4
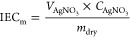
4where  (L) is the volume of the AgNO_3_ solution and  (mmol L^–1^) is the concentration of the AgNO_3_ solution.

The volumetric IEC_v_ (mequiv cm^–3^) is calculated from eq. 5
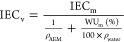
5

#### 2.3.4 Hydration Number (λ)

λ is denoted as the quantity of water molecules surrounding each cation, which is obtained by eq. 6
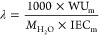
6 is the molar mass of H_2_O.

#### 2.3.5 Conductivity (σ)

The conductivity of the membranes was assessed by measuring resistances through electrochemical impedance spectroscopy (EIS). EIS data are collected on an alternating current (AC) impedance/gain-phase analyzer (AutoLab, PGSTAT204, 10^6^ to 1 Hz). The membrane is assembled in a homemade cell for collecting impedance data. The conductivities (σ, mS cm^–1^) of membranes were obtained from eq. 7
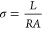
7where *L* (cm) is the distance between reference electrodes, *A* (cm^2^) is the active areas of the membrane (thickness × width), and *R* (Ω) is the resistance of the membrane.

#### 2.3.6 Diffusion Coefficient (*D*)

The *D* (cm^2^ s^–1^) of the OH^–^ can be determined by Nernst–Einstein eq. 8.^23^
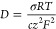
8where *z* and *F* are the valence charge and Faraday’s constant, respectively. The ion concentration, *c* (mol cm^–3^), is calculated from eq. 9
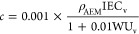
9where ρ_AEM_ and WU_v_ are the polymer density and volume-based WU, respectively.

#### 2.3.7 Maximum Ion Diffusivity (*D*_0_)

*D*_0_ (cm^2^ s^–1^) is calculated from eq. 10.^23^
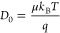
10where μ is the dilute solution hydroxide ion mobility (197.6 × 10^–5^ cm^2^ V^–1^ s^–1^),^24^*k*_B_ is the Boltzmann constant (1.38 × 10^–23^ J K^–1^), *T* (K) is the absolute temperature, and *q* is the ion charge (1.60 × 10^–19^ C).

#### 2.3.8 Alkaline Stability

The AEMs were soaked in a 1 M KOH solution at 50 °C for 1200 h, during which the chemical structure and conductivity of the AEMs were monitored.

### 2.4 Atomistic MD Simulations

#### 2.4.1 Creation of Polymeric Periodic Cells

**Figure 1:**
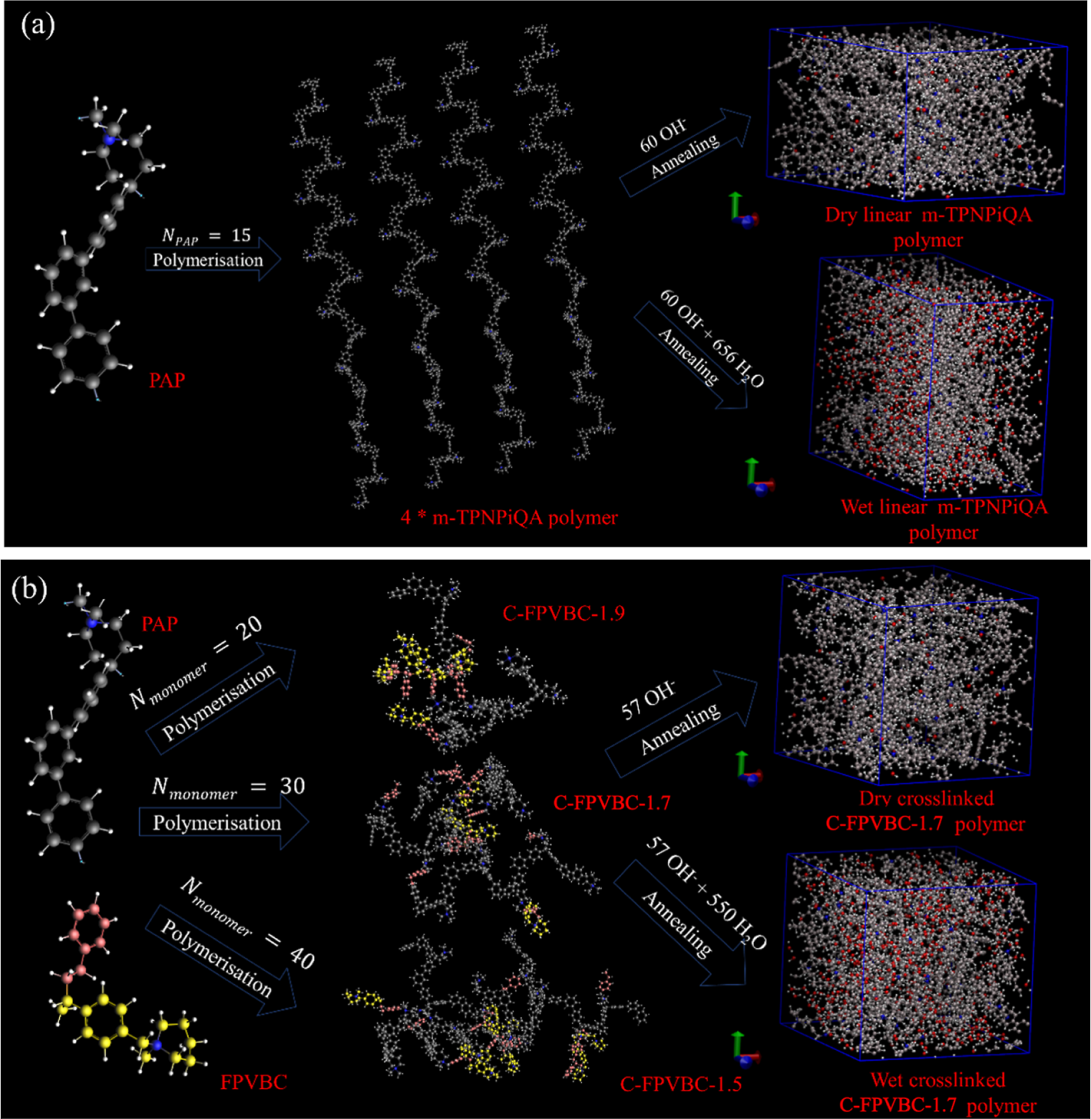
Creation of polymeric periodic cells from monomers. (a) Linear polymer cells from the polymerization of the PAP monomer and subsequent annealing. (b) Cross-linked polymer cells from cross-linking of PAP and FPVBC monomers and annealing. Color scheme of atoms: gray and pink and yellow (cross-linker)—carbon, white—hydrogen atoms, red—oxygen, blue—nitrogen.

MD simulation study was conducted on both dry and wet (with added water molecular) linear *m*-TPNPiQA polymer and cross-linked C-FPVBC-1.7 polymer. Polymerization of the PAP monomer results in a linear *m*-TPNPiQA polymer. Cross-linking and polymerization of PAP and FPVBC monomers give a C-FPVBC-*x* system. These monomers have a quaternary ammonium ion (QA) and are neutralized with an OH^–^ counterion (Figure 1).

##### 2.4.1.1 Linear *m*-TPNPiQA Polymer Systems

First, a single chain of the *m*-TPNPiQA polymer was created by polymerizing 15 PAP monomers (*N*_PAP_ = 15). Then, 4 of these linear *m*-TPNPiQA polymers were arranged along with 60 OH^–^ counterions in a cell to make a dry linear *m*-TPNPiQA polymer simulation cell. For wet linear *m*-TPNPiQA polymer system simulations, 656 water molecules (λ = 10.92 water/QA, obtained from experiments) were added along with the polymer and counterions.

##### 2.4.1.2 Cross-Linked C-FPVBC-1.7 Polymer Systems

PAP and FPVBC were polymerized and cross-linked to form cross-linked C-FPVBC-*x*, where *x* = *N*_FPVBC_/*N*_PAP_, where *N*_FPVBC_ and *N*_PAP_ are the numbers of FPVBC and PAP monomers, respectively, and *N*_monomer_ = *N*_FPVBC_ + *N*_PAP_. Three different cross-linked polymer configurations were created with *x* = 1.5 (*N*_monomer_ = 40), 1.7 (*N*_monomer_ = 20), and 1.9 (*N*_monomer_ = 30). The total number of QA for this configuration is 57 with an average *x* = 1.7. Thus, the cross-linked polymers were neutralized with 57 OH^–^ counterions to make a dry cross-linked polymer cell. To create a wet cross-linked polymer system, 550 water molecules were added with lambda equal to 9.64 water/QA (extrapolated from experiments).

#### 2.4.2 Computational and Simulation Details

MD simulations were conducted utilizing the force-field MD module of Amsterdam Modeling Suite.^25^ The systems have three entities: polymers, counterions, and water. Therefore, three force fields were used for the simulations. The polymer was described utilizing the AMBER force field, OH^–^ was described utilizing the force field proposed by Han et al., and H_2_O was described utilizing the SPC/E force field.^26^ The equations of motion were integrated utilizing the velocity Verlet algorithm.^27^ A Nose–Hoover temperature thermostat was used for isothermal–isochoric (*NVT*) simulations, and a Berendsen barostat was used for the isothermal–isobaric (*NPT*) simulations.^28^ Coulombic interactions were obtained by utilizing particle mesh Ewald summation. In contrast, nonbonded van der Waal’s interactions were truncated at a cutoff of 15 Å. Periodic boundary conditions were employed to enforce the bulk nature of the system.^29^ Four separate systems were created for the simulations: dry and wet linear (with added water molecular) *m*-TPNPiQA polymer systems and cross-linked C-FPVBC-1.7 systems.

### 2.5 Measurements of Water Electrolyzers

To evaluate the performance of the AEMWE cell, an experimental device that contains an electrochemical workstation (Autolab PGSTAT302N), two peristaltic pumps (5 mL min^–1^), and an electrolysis cell (Dioxide materials, USA) with a corrosion-resistant nickel bipolar plate and serpentine flow channels was assembled. Membrane electrode assembly (MEA) was prepared from an anode (the NiFe_2_O_4_ catalyst was loaded on a stainless-steel gas diffusion layer, US Research Nanomaterials), a C-FPVBC-1.7 AEM, and a cathode (NiFeCo alloy nanoparticles loaded onto nickel fiber paper). Each electrode was loaded with a catalyst of 2 mg cm^–2^. Before assembly, the AEM was kept in a 1 M KOH solution overnight. Subsequently, the membrane is positioned between the cathode and anode electrodes, and a hot-pressing is applied at 10 MPa at 50 °C for 10 min to produce an MEA (with an active area of 1.0 cm × 1.0 cm). The temperature of the electrolyte is adjusted by a water bath. The operating temperature of the electrolysis cell was regulated using a thermocouple and a PID controller.

The cell was initialized for a period of 0.5 h at a current density (10–50 mA cm^–2^). Polarization curves were generated by sweeping the potential within the range of 1.5 to 2.6 V at a scanning rate of 5 mV s^–1^ under ambient conditions, as well as at temperatures of 50 and 80 °C. The impedance measurements were performed under a constant voltage (1.5–2.1 V). The durability of the electrolyzer was carried out at a current density of 100 mA cm^–2^ (50 °C).

## 3 Results and Discussion

### 3.1 Preparation of C-FPVBC-*x* AEMs

PAP was synthesized by a typical polycondensation reaction catalyzed by acid.^30^Figure S1 illustrates the ^1^H NMR spectrum of PAP. The signals detected in the 7–8 ppm range are assigned to the protons associated with the arylene groups. The signals (H_1_) from the piperidine group split into two peaks (2.34 and 2.85 ppm), a result of the protonation of piperidine.^31^ The peak from 2.75 ppm originates from the methyl proton (H_3_). The integral ratio of H_3_ to H_1_ in the PAP polymer is 1.00:1.47, which matches the theoretical value of 1:1.50. As depicted in Figure S2 (FPVBC), the signals (H_3,4,10,11_) from 6.50 to 7.50 ppm are assigned to protons from arylene groups. The resonance at 3.01 ppm (H_6_) is assigned to −CH_3_ within piperidinium groups. The proportion of the integral area corresponding to the peak at 3.01 ppm to that of the peak at 4.74 ppm (H_5,12_) aligns with the expected theoretical values. These results indicate the successful synthesis of PAP and FPVBC.

C-FPVBC-*x* membranes (60 ± 5 μm) with *x* > 1.7 exhibited an inward curling and increased brittleness (Figure S3). Hence, we did not explore the performance of AEMs with *x* > 1.7. The digital photo (Figure S4) and SEM images (Figure S3) of the C-FPVBC-1.7 membrane showed that rigid, transparent, and dense AEMs were prepared. Table 1 and Figure S5 show the GF results of the cross-linked C-FPVBC-*x* AEMs. It is observed that GF increased with the rising ratio of FPVBC, reaching a peak value of 89.15%.

**Table 1:** GF, IEC, WU, SR, Conductivity, and *D*_OH_^–^ of C-FPVBC-*x* Membranes at 20 °C

AEMs	GF (%)	IEC_theo_ (mequiv g^–^^1^)^a^	IEC_m_ (mequiv g^–^^1^)^b^	IEC_v_ (mequiv cm^–^^3^)^c^	WU_m_ (%)^b^	WU_v_ (%)^c^	SR (%)	λ (OH^–^)	σ (mS cm^–^^1^)	*D*_OH_^–^ (10^–^^9^ cm^2^ s^–^^1^)^c^
C-FPVBC-0.7	78.08	3.20	2.82	2.69	23.64	29.19	3.98	4.66	15.68	0.48
C-FPVBC-0.8	82.36	3.21	2.85	2.53	32.24	40.09	5.97	6.46	28.78	1.00
C-FPVBC-1.0	85.29	3.23	2.89	2.48	39.12	50.53	8.00	7.50	31.39	1.16
C-FPVBC-1.2	87.18	3.25	2.98	2.49	44.54	59.14	14.90	8.68	36.50	1.38
C-FPVBC-1.7	89.15	3.28	3.19	2.50	52.65	70.09	20.56	9.17	40.15	1.61
*m*-TPNPiQA^d^		2.66^d^	2.54	2.09	52.28	73.27	21.14	10.92	22.11	1.04

a Calculated from the feed ratio.

b Determined by titration.

c Calculated from IEC_m_.

d Obtained from ref. (16).

**Figure 2:**
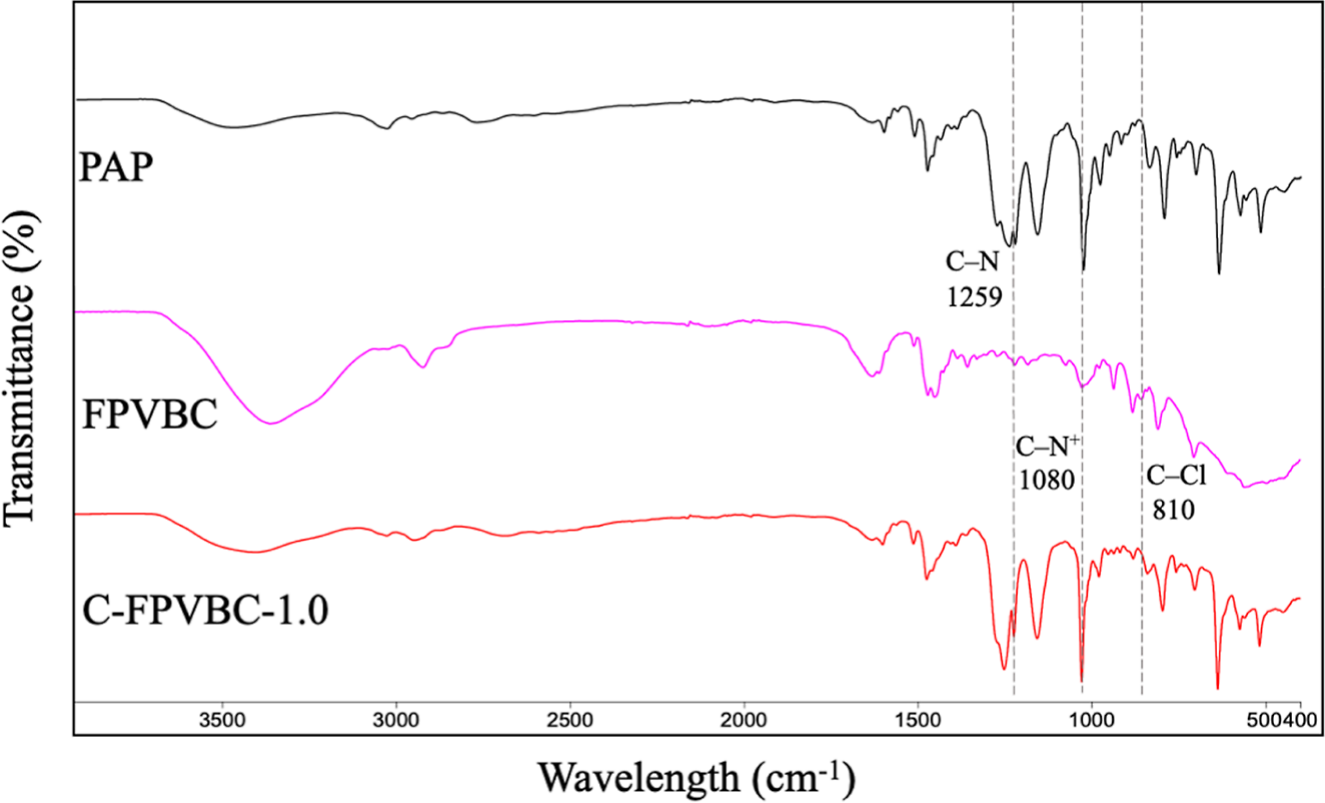
FT-IR spectra of the precursor and the membrane.

Figure 2 demonstrates the FT-IR spectra of C-FPVBC-*x* membranes. The absorption bands at 1647, 1497, and 1465 cm^–1^ are associated with the distinctive characteristics of the arylene groups.^32^ Additionally, the features close to 1465 cm^–1^ are ascribed to the stretching vibration and deformation vibration of C–H (piperidinium group).^30^ The bands at 3026 and 2929–2852 cm^–1^ belong to the stretching vibrations of aromatic C–H and alkane C–H stretching, respectively.^33^ Moreover, distinctive signals from C–N^+^ are evident at approximately 1080 cm^–1^.^33^ After the cross-linking reaction, the intensity of the peak at 1259 cm^–1^, corresponding to the signals from C–N, is diminished owing to the incorporation of cations. The disappearance of the characteristic C–Cl stretching peak at 810 cm^–1^ in FPVBC indicates the successful cross-linking reaction between PAP and FPVBC chains.^30^ As exhibited in Figure S6, the intensity of signals (3400 cm^–1^) from OH^–^, attributed to H_2_O, exhibited an increment with the increasing FPVBC content.^34^

### 3.2 Membrane Morphology

**Figure 3:**
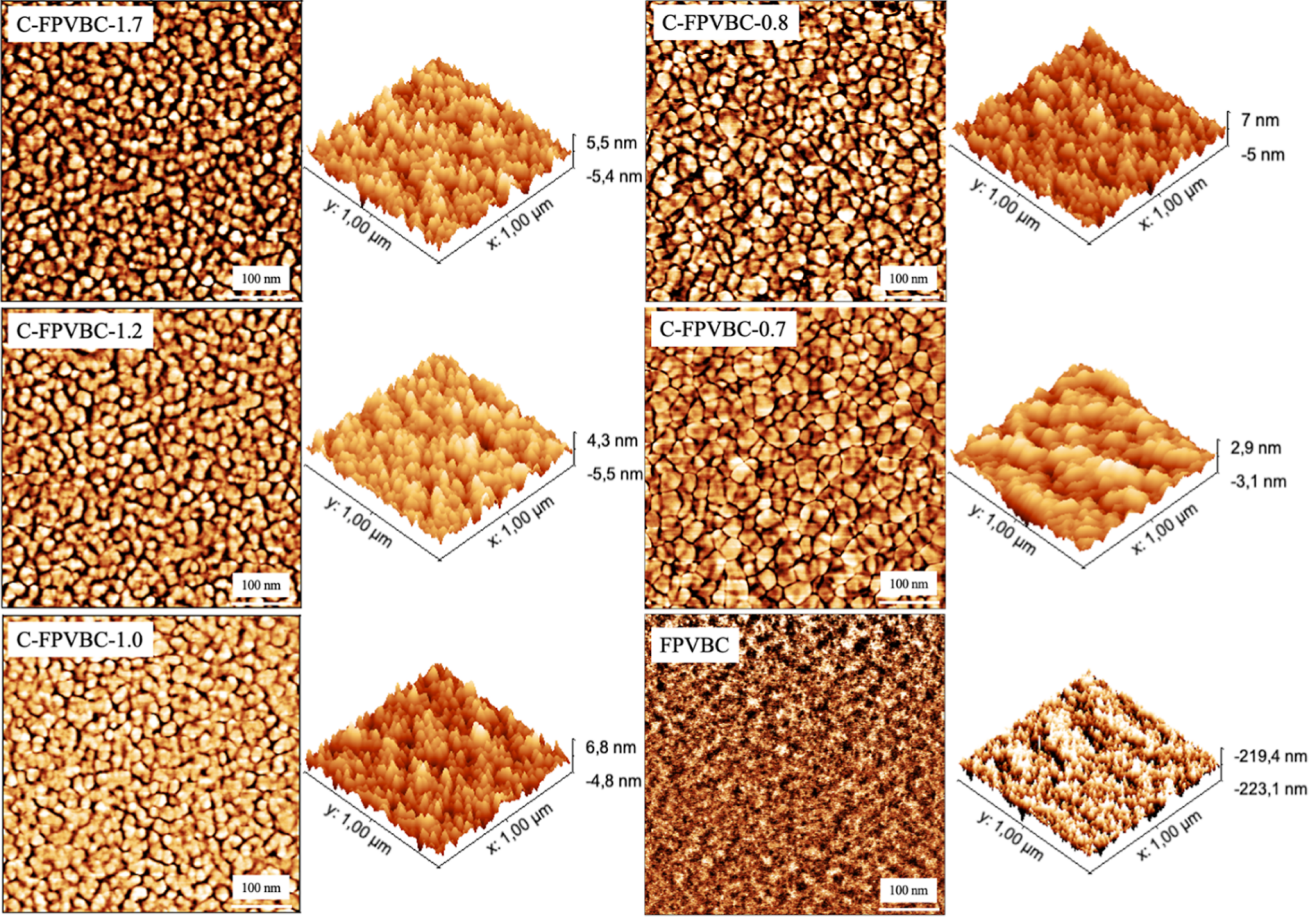
AFM images of the polymer and membranes.

AFM and SAXS were utilized to explore the microstructures of the membranes. It is pointed out that the thermodynamic incompatibility of the hydrophobic/hydrophilic region could drive the membranes to form microphase separation. As illustrated in Figure 3, the darker sections correspond to the hydrophilic region, which primarily consists of cations and H_2_O. The bright sections represent the hydrophobic region, predominantly constituted by polymer chains of poly(phenylene) and FPVBC.^35^ Interestingly, the differences in the chain entanglement structure of FPVBC and PAP result in various morphologies for membranes. The higher the concentration of FPVBC, the more pronounced the obvious hydrophobic/hydrophilic phase separation. The agglomeration of piperidinium cations results in large ionic clusters. By comparison, the functionalized FPVBC polymer membrane has featureless or virtually no appreciable ion clusters, similar to *m*-TPNPiQA.^20^ The results indicated that a well-connected ion conduction pathway forms in the C-FPVBC-1.7 membrane, allowing for highly efficient ion conduction and ultimately enhancing the conductivity of the membrane.

**Figure 4:**
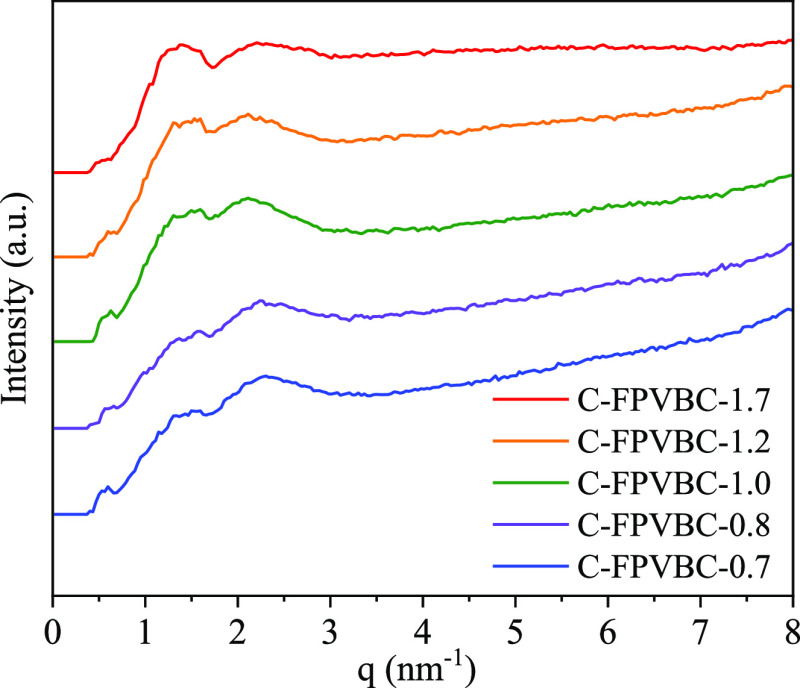
SAXS plots of the C-FPVBC-*x* AEMs.

Figure 4 illustrates the SAXS plot of the AEMs. The characteristic peak *q* values align with the ionomer peak, which arises from the existence of ion-rich domains.^36^ The C-FPVBC-*x* exhibits two clear peaks (1.3 and 2.1 nm^–1^). C-FPVBC-1.7 with a higher IEC value shows a more substantial peak at 1.3 nm^–1^. Utilizing the Bragg equation (*d* = 2π/*q*), the calculated *d*-spacing for these peaks is 10.47 and 4.83 nm, respectively, consistent with the AFM results. A higher *d*-spacing value indicates larger hydrophilic (ion-rich) domains, forming efficient ion transport pathways.

### 3.3 IEC, WU, and SR

As demonstrated in Table 1, the IEC_theo_ values of AEMs exhibited a slight rise with an increase in the FPVBC content. When hydrated, the IEC_v_ could reflect the ion pair concentration within the ionomer matrix.^37^ The IEC_v_ of C-FPVBC-*x* AEMs decreased with increasing cross-linking degree. Excessive water inside the membrane is a probable factor contributing to the lower IEC_v_ value of *m*-TPNPiQA, which could lead to reduced hydroxide conductivity. A comparable occurrence is evident in other literature.^38^

AEMs rely on water molecules to facilitate ion-conducting pathways, which means that the essential role of the membrane’s water absorption capability in sustaining high conductivity. The water absorption characteristics of membranes are predominantly influenced by the IEC and the intramolecular and intermolecular structures within the AEMs. As displayed in Figure S7, the WU and SR of C-FPVBC-*x* membranes align with the cross-linking degree and IEC_m_ values. In particular, the C-FPVBC-1.7 membrane, with an IEC_m_ of 3.19 mequiv g^–1^, exhibits the highest WU of 74.73% and SR of 25.42% at 80 °C. These values surpass those of the C-FPVBC-0.7 (WU = 35.21%, SR = 8.44%). Both the cross-linking degree and the IEC value exert an influence on the WU and SR. Although the theoretical IEC (IEC_theo_) values of C-FPVBC-*x* (*x* = 0.7 and 0.8) are similar (3.20 vs 3.21 mequiv g^–1^), their IEC_v_ values differ (2.69 vs 2.53 mequiv g^–1^). Consequently, the WU and SR increase with increasing IEC_v_ values. C-FPVBC-1.7, exhibiting the highest cross-linking degree, demonstrated the highest WU and SR. This is primarily ascribed to the elevated IEC_m_ value (2.82 to 3.19 mequiv g^–1^). An analogous occurrence has been found in the literature.^39^ Moreover, the WU and SR of the C-FPVBC-*x* membranes exhibited an upward trend with a rising temperature. However, the swelling behavior of AEMs results in membrane wrinkling, potentially leading to delamination of both the membrane and catalyst layers in AWMWE.^40^ A limited SR could be achieved in AEMs with cross-linked structures.^41,42^ The C-FPVBC-*x* membranes exhibit decreased SR at comparable IEC_m_ and temperature compared to some previously reported AEMs, indicating that the cross-linking structure efficiently enhances the dimensional stability of the AEMs.^42,43^

### 3.4 Hydroxide Conductivity

**Figure 5:**
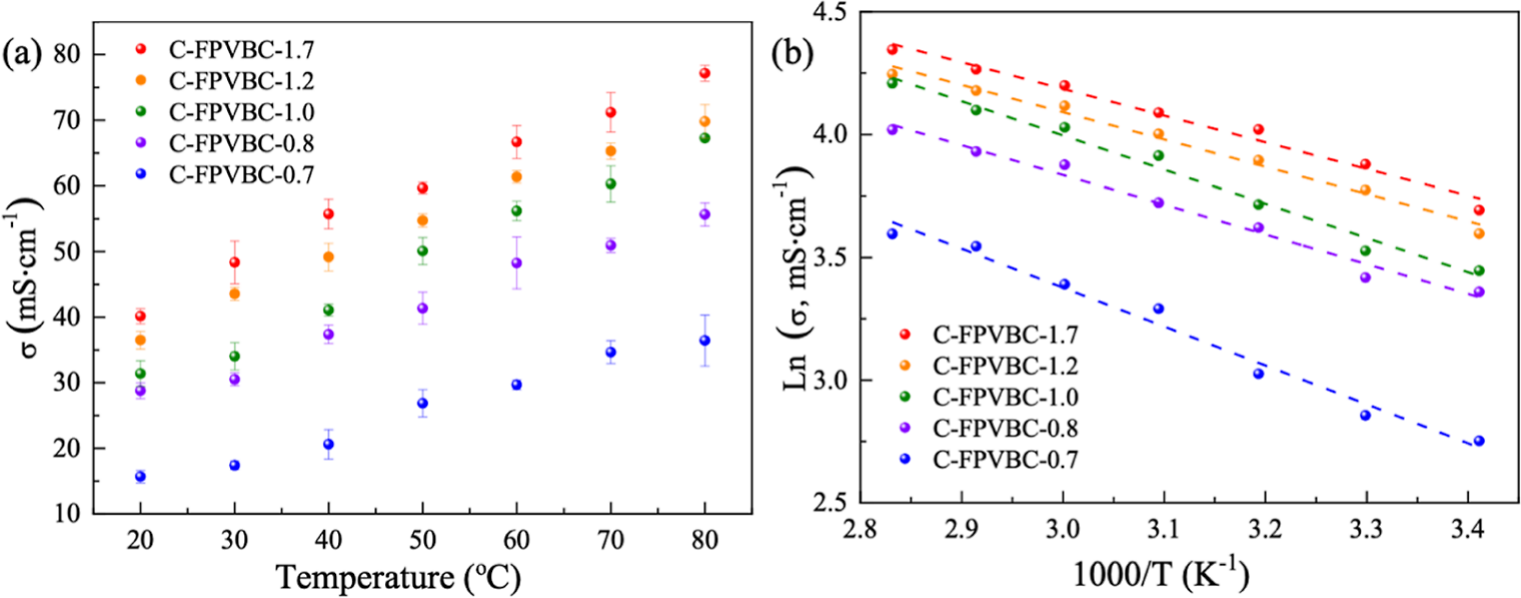
Hydroxide conductivity (a) and corresponding Arrhenius plots (b) of AEMs.

The hydroxide conductivity is crucial for attaining high performance for the water electrolyzer. To minimize the ohmic resistance of an electrolyzer cell, AEM_S_ should exhibit high ionic conductivity. As illustrated in Figure 5a, the conductivity showed an upward trend as the IEC increased. The C-FPVBC-1.7 membrane with an IEC_m_ of 3.19 mequiv g^–1^ demonstrates the highest conductivity, reaching 77.15 mS cm^–1^ at 80 °C, nearly double that of the C-FPVBC-0.7 membrane (36.44 mS cm^–1^). AFM results (Figure 3) indicated that the C-FPVBC-0.8 membrane exhibited a more distinct hydrophobic/hydrophilic phase separation than C-FPVBC-0.7 due to the increased cross-linking degree that facilitates the aggregation of ionic groups. This resulted in an increased conductivity for C-FPVBC-0.8. With an increase in IEC_m_, the conductivities of C-FPVBC-*x* exhibit reduced sensitivity to changes in temperature. A moderate water content of the C-FPVBC-1.7 membrane facilitates the transport of OH^–^. The optimal proportion of FPVBC and PAP is important for improving the mobility of the cationic groups, thereby generating additional space for effective ion conduction. As a result, the conductivity of C-FPVBC-*x* membranes exhibited an increase in tandem with the ratio of FPVBC to PAP. The efficient ion transportation is ascribed to the fabrication of the ionic domain through cross-linking, which creates an effective “ionic highway” for ion conduction, as shown in Figures 3 and 4. Here, we introduce conductivity (σ) against the IEC to assess the features of the AEMs. A higher σ/IEC of AEMs correlates with an elevated conductivity under the same IEC. As indicated in Table S4, the σ/IEC of C-FPVBC-1.7 is 15.1 at 30 °C, significantly surpassing that of *m*-TPNPiQA (σ/IEC = 10.2). Furthermore, a comparison with other reported poly(arylene piperidinium)-based AEMs is also illustrated. The σ/IEC of C-FPVBC-1.7 exceeds that of some reported AEMs based on poly(arylene piperidinium), highlighting the distinctive features of the C-FPVBC-*x* membranes.

Figure 5b illustrates the temperature-dependent conductivity of C-FPVBC-*x* membranes, which is portrayed using an Arrhenius-type representation, with the activation energy (*E*_a_) calculated within the range of 9.02–13.19 kJ mol^–1^. The calculated *E*_a_ values were similar to or lower than those reported for other AEMs (10–17 kJ mol^–1^),^16,44^ as well as previously reported *m*-TPNPiQA AEM (15.4 kJ mol^–1^), demonstrating that cross-linked AEMs exhibit a similar ion conducting mechanism to the reported AEMs.

**Figure 6:**
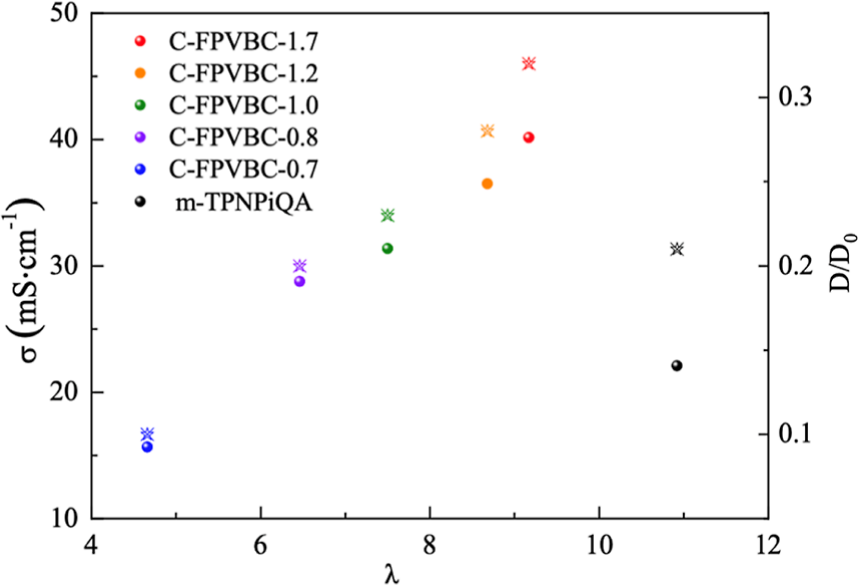
Conductivity (colored circle) and *D*/*D*_0_ (stars) of AEMs at 20 °C.

Figure 6 illustrates the relationship between the hydroxide conductivity and λ of C-FPVBC-*x* membranes. It is found that C-FPVBC-*x* AEMs (λ = 4.66–9.17) demonstrate lower λ compared to TPNPiQA. Among them, C-FPVBC-1.7 (λ = 9.17) displays the highest hydroxide conductivity. In contrast, the *m*-TPNPiQA (λ = 10.92) showed lower conductivity attributable to diluted concentration of ionic groups.^16^ Normalized diffusion coefficient (*D*/*D*_0_) is determined by comparing the diffusion coefficient of an AEM (OH^–^ form) to the maximum diffusivity of hydroxide ions in water (*D*_0_). *D*/*D*_0_ is employed to compare the ion conduction in wet membranes. A high *D*/*D*_0_ results in enhanced mobility of the hydrated OH^–^ and improved conductivity.^45^Figure 6 displays the correlation between *D*/*D*_0_ and λ of the membranes. C-FPVBC-*x* (*x* = 1.2 and 1.7) exhibited the highest *D*/*D*_0_ values of 0.32 (λ = 9.71) and 0.28 (λ = 8.68), respectively, surpassing those of *m*-TPNPiQA (λ = 0.21). This occurs due to the incorporation of FPVBC, which offers additional active sites facilitating ion conduction, leading to a more effective pathway for ion transport.^46^ The incorporation of a macromolecular cross-linker into the membranes allows for a higher concentration of cations and reduces λ, thereby enhancing conductivity.

### 3.5 MD Simulations

**Figure 7:**
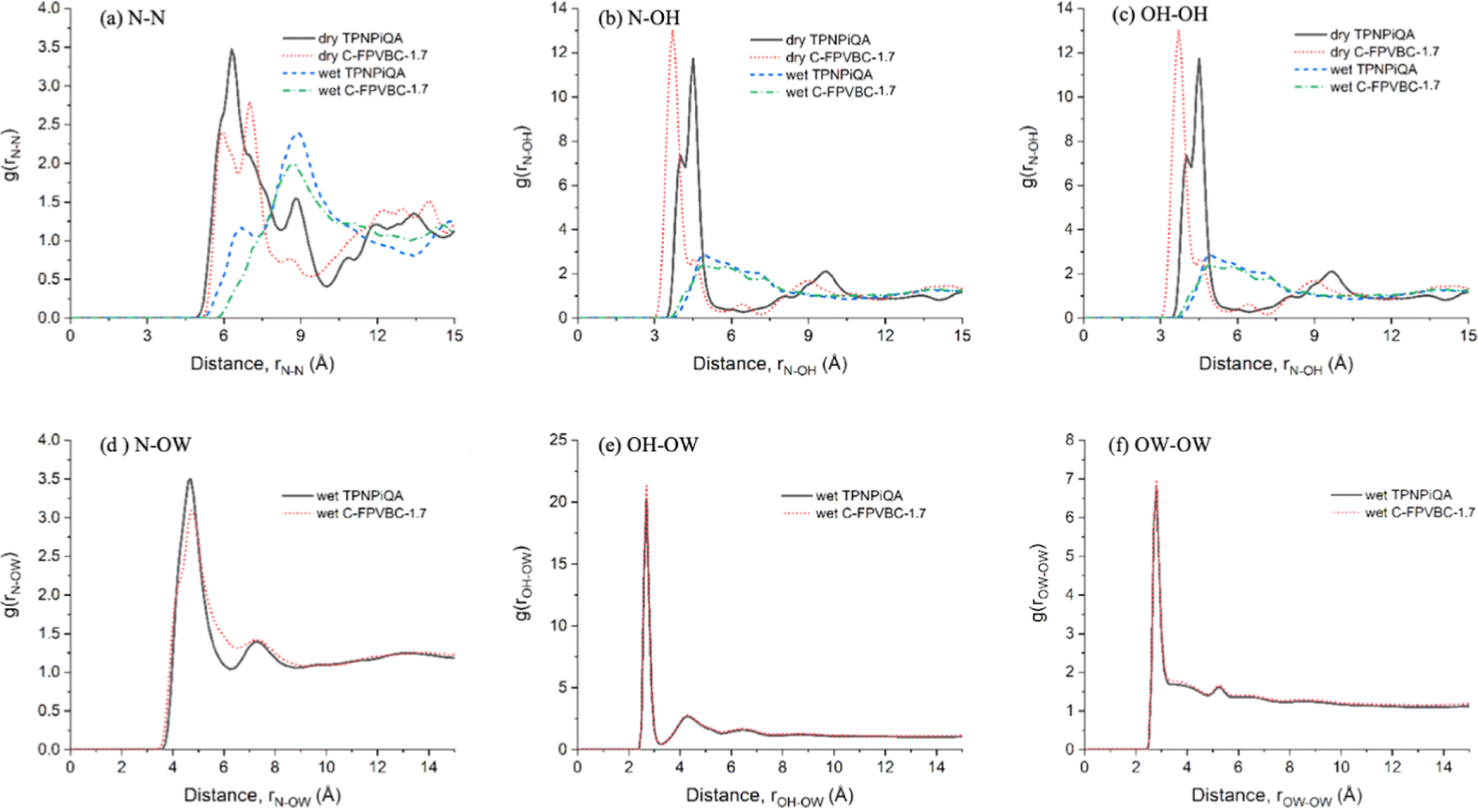
Radial distribution function of atomic species: (a) N–N, (b) N–OH, (c) OH–OH, (d) N–OW, (e) OH–OW, and (f) OW–OW in the dry and wet polymer systems.

MD simulations were carried out to examine how the microstructure affects the performance of AEMs. Figure 1 shows the model of cells. The radial distribution function is calculated for the linear *m*-TPNPiQA and cross-linked C-FPVBC-1.7 in dry and wet conditions, as shown in Figure 7. The first coordination shell radius, *r*_A–B_^max^ (Å), and the coordination number are tabulated in Table S2. The pair correlation function *g*_A–B_(*r*) analysis was performed to understand the atom distribution within the polymer and the specifics of packing. When water is added to both polymer systems, the first coordination shell radius between two N atoms and between an N atom and an OH^–^ increases, whereas the nearest OH–OH distance decreases. This means water additions reduce the polymer–polymer and polymer–hydroxyl interactions and cause an OH–OH clustering. It can also be seen that hydroxyl atoms get a tighter hydration shell (OH–OW) than the polymer (N–OW), which can affect the hydroxyl group dynamics. However, the difference between the linear and cross-linked polymers is minimal as they appear to show similar interactions with hydroxyl and polymer.

**Figure 8:**
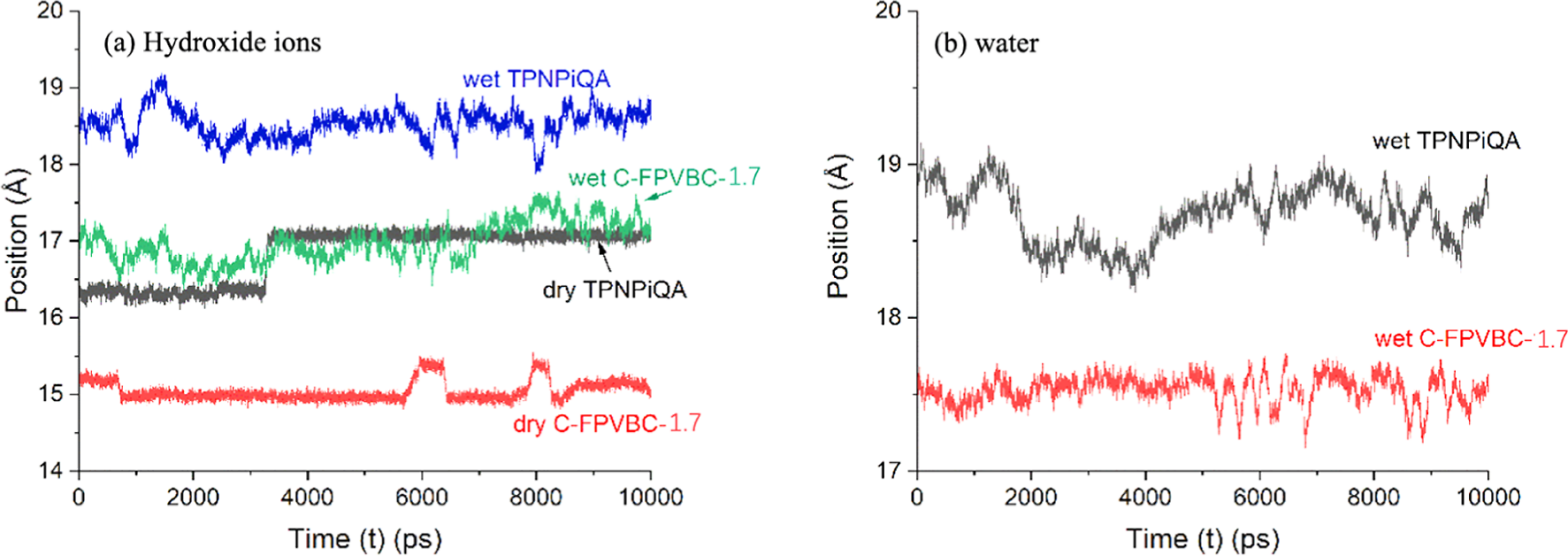
Positions of (a) hydroxide ions and (b) water centers in the polymer systems.

The positions of the hydroxide and water species are demonstrated in Figure 8. The hydroxide ion jumps found in the dry system are completely diminished with the presence of water as the ions become more mobile throughout the system. To better understand this mobility, the trajectories achieved from MD simulations were employed to calculate the mean square displacement of the species and their diffusivity (Table S3). Then, from Einstein’s equation for Brownian motion, self-diffusivity is calculated as . The trends in diffusivity values obtained from the simulations also confirm that the cross-linking increases the overall rate of hydroxyl ion diffusion. The calculations predict that the *D*_OH_^–^ value in C-FPVBC-1.7 is 8.53 × 10^–9^ cm^2^ s^–1^, five times higher than that in *m*-TPNPiQA (1.65 × 10^–9^ m^2^ s^–1^). This causes a slight mismatch with the experimental predictions. This arises because the simulations create a defect-free environment for the hydroxyl ions, which is nearly impossible to obtain in experiments.

### 3.6 Mechanical Properties and Thermal Stability

The limited mechanical strength of currently designed AEMs impedes their practical applications.^47^ Ensuring robust mechanical properties in AEMs is vital to impede damage or rupture during MEA fabrication and to inhibit H_2_ permeation throughout the water electrolysis process. AEM with high elongation at the break exhibits flexibility, allowing the membrane to withstand stress before it cracks.^48^ For mechanical property measurements, the AEMs are tested in a hydrated condition (wet membrane). As depicted in Figure S8, the tensile strengths of C-FPVBC-*x* membranes varied between 5.67 and 25.45 MPa, while the elongation at break ranged from 9.66 to 16.69%. A higher cross-linking degree could lead to a lower elongation at break.^49^ Thus, the AEMs exhibited a reduction in elongation at break with an increase in cross-linking degree. Water can act as a plasticizer and enhance elongation at the break of the membranes.^50^ C-FPVBC-1.2 exhibited higher WU than C-FPVBC-1.0. Thus, C-FPVBC-1.2 demonstrates a higher elongation at break compared to C-FPVBC-1.0. In contrast, the fabrication of a cross-linking structure, achieved through the combination of the PAP and FPVBC, results in the cross-linked AEMs exhibiting comparable or superior performances compared to PAP- or FPVBC-based AEMs, as indicated in Table S5. The tensile strength of the AEMs exhibited an upward trend with an increasing ratio of PAP. The lower cross-linking degree decreases the tensile strength due to the lower WU and water plasticization.^51^ The C-FPVBC-1.7 AEM exhibited the highest tensile strength (25.45 MPa) and an elongation at break (16.69%). The improvement in mechanical properties could be ascribed to the disruption of the semicrystalline characteristic of FPVBC and PAP polymers achieved through cross-linking. A similar phenomenon was observed in other cross-linked membranes reported in the literature.^52^ The Sustainion 37-50 exhibits susceptibility to cracking in a dry state, highlighting the suboptimal mechanical properties of the pure FPVBC.^40^

**Figure 9:**
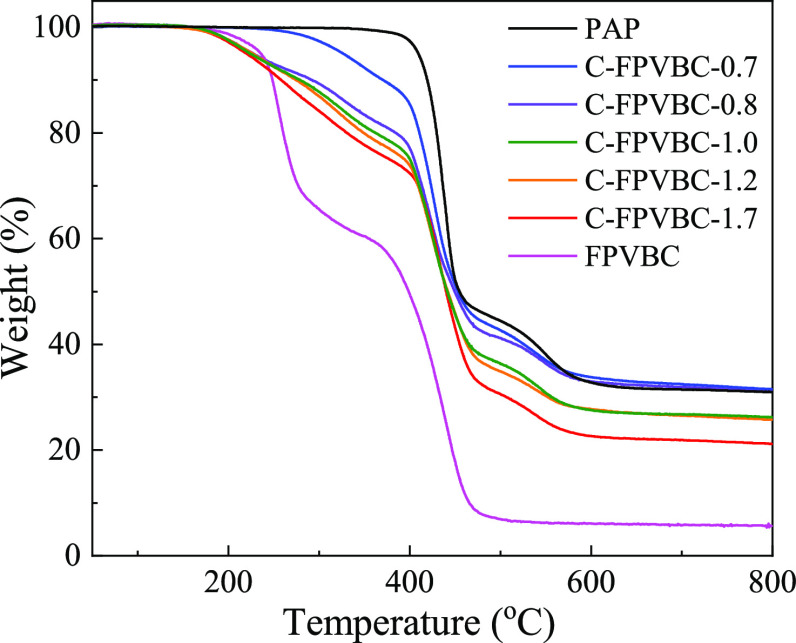
TGA of the precursor polymer and C-FPVBC-*x*.

TGA of the precursor and C-FPVBC-*x* was conducted (see Figure 9). The PAP polymer exhibited stability without undergoing decomposition up to 400 °C. The C-FPVBC-*x* AEMs showed a marginal decrease in weight within the temperature range of 180–380 °C, possibly due to the decomposition of the cations and alkyl chains.

### 3.7 Alkaline Stability

**Figure 10:**
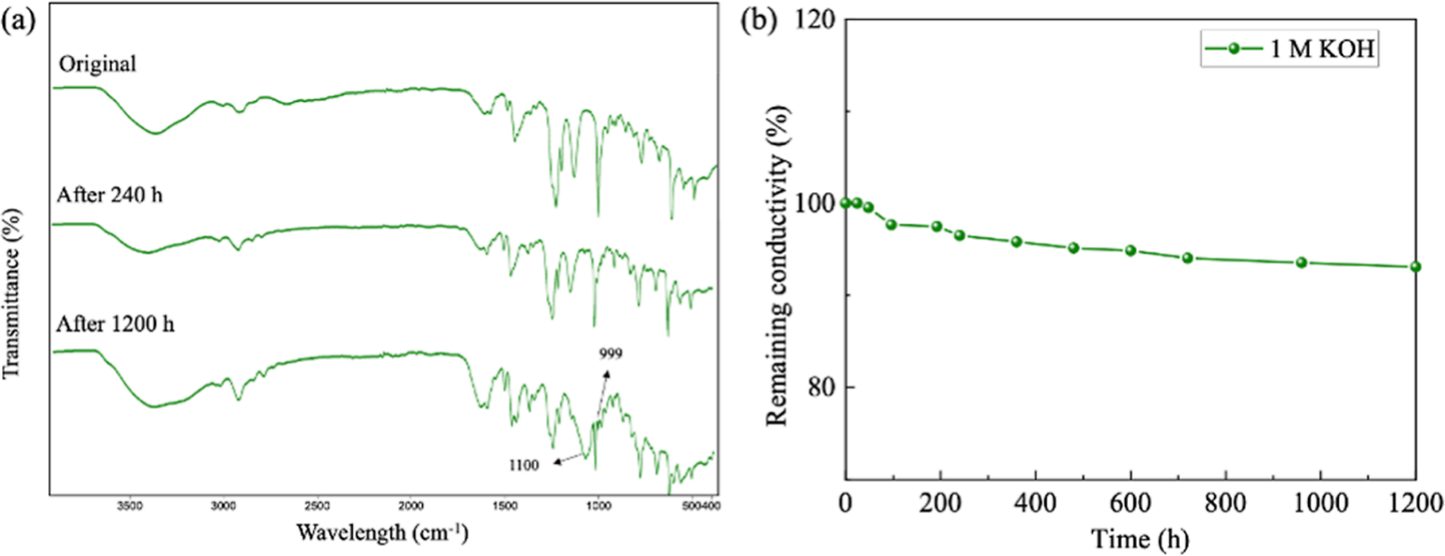
(a) FT-IR spectra of the C-FPVBC-1.7 membrane (1 M KOH, 50 °C) and (b) remaining conductivity of the C-FPVBC-1.7 membrane.

The degradation of AEMs under alkaline environments predominantly arises from cation degradation through processes such as Hofmann elimination, the formation of ylide intermediates, nucleophilic substitution reactions, and/or chemical rearrangement.^53^ There are some reports about the stability of polyethylene backbones and piperidinium groups under alkaline conditions.^20,54^ To gauge the practical stability of the AEM in a water electrolyzer and consolidate the results for comparison, the alkaline stability test is conducted at 50 °C. The chemical structure of the AEMs was investigated using FT-IR before and after exposure to 1 M KOH (1200 h at 50 °C), as illustrated in Figure 10a. The distinctive peaks (1259, 1080 cm^–1^), attributed to the stretching vibration of C–N and C–N^+^, exhibited minimal changes. The peaks within the 2854–2956 cm^–1^ range associated with aliphatic C–H stretching vibration became prominent, suggesting that the principal degradation of the cations likely occurred through a ring-opening reaction.^45^ The slight intensities of the peaks observed at 1420 and 1600 cm^–1^ can be ascribed to the stretching vibration of the C=C bonds in aromatic rings. The peaks observed at 1635 and 1380 cm^–1^ were associated with the stretching and bending vibrations of the olefin groups. The appearance of additional peaks at 1100 and 999 cm^–1^ can be attributed to the C–H deformation vibration within the olefin groups. The degradation of the C-FPVBC-1.7 AEM primarily resulted from the Hofmann elimination, as confirmed by other researchers.^30^

The plot of residual conductivity against an immersion time is presented in Figure 10b. The conductivity retained 96.48 and 93.1% of its original value after 240 and 1200 h, respectively. The outstanding stability under alkaline conditions can be attributed to the geometric constraints imposed by the piperidinium group, which hinder the relaxation of ring strain, thereby reducing the activation energy of degradation reactions.^55^ Simultaneously, the cross-linked structure decreased the likelihood of OH^–^ attacking the polymer backbone or cations. This is attributed to the lack of aryl-ether bonds, contributing to an improved stability in alkaline condition.^5^ The findings indicate that the cross-linked C-FPVBC-*x* exhibits robust chemical stability when subjected to alkaline conditions.

### 3.8 AEMWE Performance

**Figure 11:**
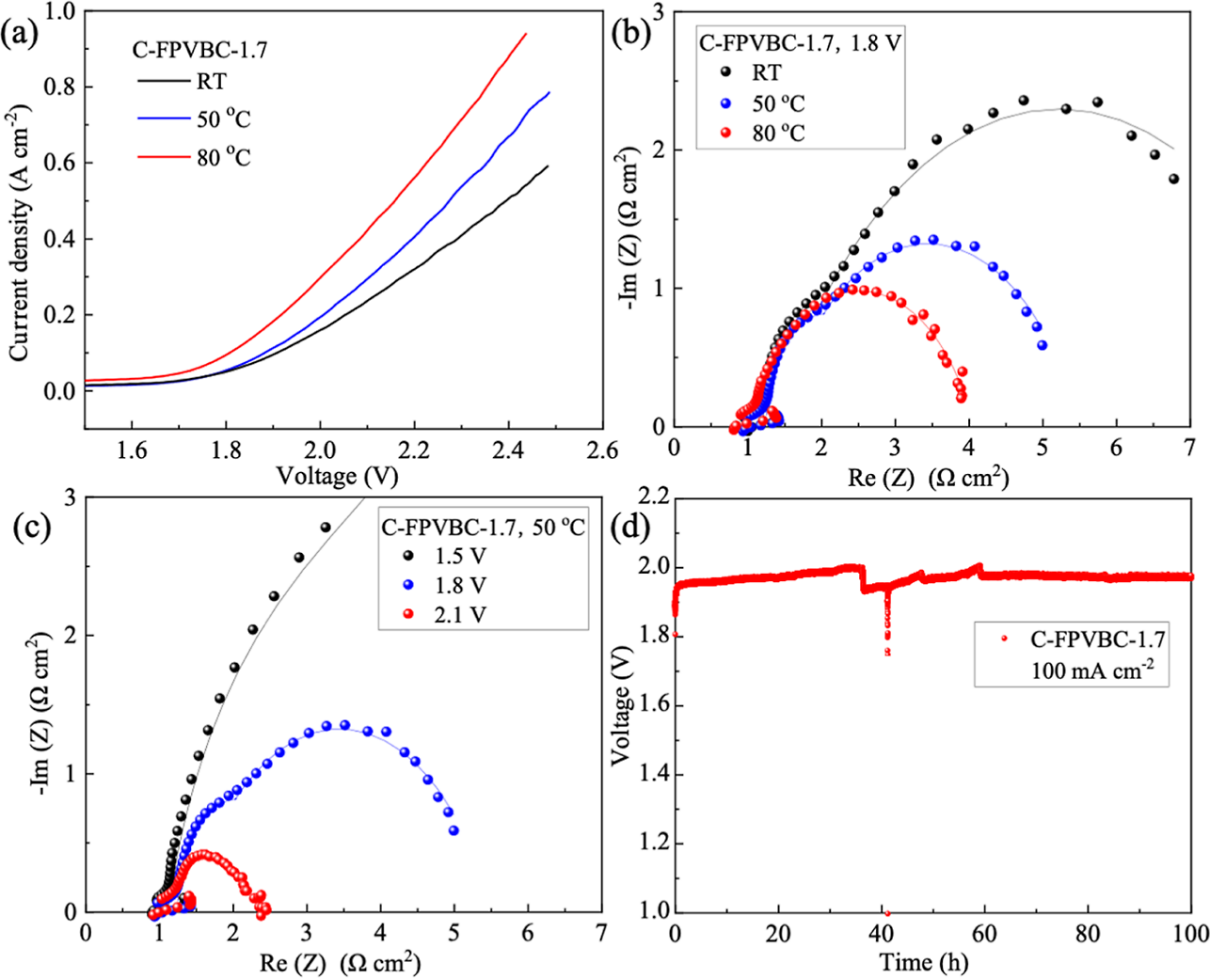
(a) Polarization curves, (b) EIS spectra, and (c) EIS spectra of the C-FPVBC-1.7-based electrolyzer. (d) Durability test of a water electrolyzer. Electrolyte: 1 M KOH (50 °C), current density: 100 mA cm^–2^, flow rate: 5 mL min^–1^.

The as-prepared C-FPVBC-1.7 membrane with the highest ionic conductivity and excellent comprehensive performance was assembled into an AEMWE instrument for measurements of water electrolysis performance. Figure 11a shows the polarization curves of AEMWE by applying a C-FPVBC-1.7 membrane. At 2.4 V, the C-FPVBC-1.7 AEMWE achieved a maximum current density of 890 mA cm^–2^ at 80 °C, 675 mA cm^–2^ at 50 °C, and 507 mA cm^–2^ at RT. Moreover, as the temperature rises, the mobility of hydroxide ions within the AEM is enhanced, leading to a faster transfer rate. The current density gradually increases with voltage, thereby accelerating the electrode reaction kinetics. The slope of the polarization curve in the high current gradually diminishes, signifying a reduction in ohmic impedance attributed to the improved conductivity of C-FPVBC-1.7 AEM at elevated temperatures. Table S6 offers a comparative analysis of the water electrolysis performance of membranes from the literature. The C-FPVBC-1.7 membrane, utilizing nonplatinum group metal catalysts, demonstrated comparable performance to some reported AEMs using platinum-based catalysts. These findings suggest that the C-FPVBC-*x* membrane has the potential for use in water electrolysis.^56,57^

Figure 11b,c illustrates the Nyquist plots of the C-FPVBC-1.7-based water electrolyzer. The corresponding equivalent circuit diagram is analyzed, and the relevant parameters and error analysis are shown in Table S7. As the temperature increased, both *R*_m_ (mass-transfer resistance) and *R*_ct_ (charge-transfer resistance, mainly in the anode compartment) of the AEMWEs decreased. This aligns with the observed trend in the polarization curves presented in Figure 11a. As the temperature rose (RT to 80 °C), the *R*_m_ reduced from 0.92 to 0.84 Ω cm^2^, and the *R*_ct_ decreased from 10.89 to 3.08 Ω cm^2^. Thus, increasing the operating temperature is efficient in improving the performance of AEMWE. For the C-FPVBC-1.7-based water electrolyzer at 50 °C, the *R*_m_ was 0.91 Ω cm^2^ at 2.1 V, 0.90 Ω cm^2^ at 1.8 V, and 0.92 Ω cm^2^ at 1.5 V. The gas production rate in the cell showed a gradual increase with the increase in voltage within the testing voltage range. A decrease in the arc diameter corresponded to reduced charge-transfer impedance, suggesting improved mass transfer of the C-FPVBC-1.7-based electrolyzer at 2.1 V.

The electrolyzer based on C-FPVBC-1.7 was operated continuously at a current density of 100 mA cm^–2^ for 100 h at 50 °C. As shown in Figure 11d, during the first 38 h, the cell voltage experienced occasional increments, reaching from 1.95 to 2.00 V. This may be attributed to the detachment of a small amount of catalyst under the flushing of the flowing electrolyte, increasing the cell impedance. The sudden decline in voltage, accompanied by intermittent minor fluctuations, is a consequence of disruptions arising from the ongoing accumulation and the release of gas produced during the electrode reaction.^58^ After 60 h, the cell voltage was maintained at 1.96 V, manifesting the excellent stability of C-FPVBC-1.7-based AEMWE. It should be mentioned that the preparation method of the MEA and the operating condition of the electrolyzer should be further optimized to achieve good water electrolysis performance.

## 4 Conclusions

In summary, novel macromolecular cross-linked C-FPBVC-*x* AEMs were prepared. In contrast to the linear *m*-TPNPiQA, the cross-linked AEMs offer a notable benefit by creating a microphase-separation structure, aligning with the findings from SAXS and AFM. The C-FPVBC-1.7 membrane demonstrated the highest conductivity, reaching 77.15 mS cm^–1^ at 80 °C. This suggests the effective establishment of an ionic pathway within the AEM. In addition, the C-FPVBC-1.7 AEM exhibited remarkable durability under prolonged exposure to alkaline conditions at 50 °C, with a mere 6.9% reduction in conductivity observed after an alkaline treatment for 1200 h. The C-FPBVC-1.7 membrane with the highest OH^–^ conductivity was assembled in an electrolyzer, which provided a high current density of 890 mA cm^2^ at 2.4 V at 80 °C. After operating for 100 h in an electrolyzer, almost no performance loss was observed for the electrolyzer based on C-FPVBC-1.7. These findings showcase the potential applicability of the cross-linked C-FPVBC-*x* membranes in practical use for AEMWE.

## Supporting Information Available

^1^H NMR spectra of all the as-synthesized products; photo of polymers and membranes; SEM image of AEM; FT-IR spectra of C-FPVBC-*x* AEMs; IEC, WU, SR, and conductivity of the C-FPVBC-*x* AEMs; mechanical and single-cell durability performance and its comparisons; computational and simulation details; and first coordination shell properties and diffusivity eco-efficiency (PDF)
