# Supplementary material for: Dimensionally Stable Anion Exchange Membranes Based on Macromolecular-Cross-Linked Poly(arylene piperidinium) for Water Electrolysis
Source: ACS Appl Mater Interfaces. 2024 Jan 4;16(2):2593–605. doi: 10.1021/acsami.3c13801 (PMC10797592; doi:10.1021/acsami.3c13801)
Supplement: Supplementary file 1 — am3c13801_si_001.pdf [file am3c13801_si_001.pdf]

Supporting Information

Dimensionally Stable Anion Exchange Membranes Based on Macromolecular-Crosslinked Poly(Arylene Piperidinium) for Water Electrolysis

Xiuqin Wang,[a,b] Angela Mary Thomas,[a,c] Rob G. H. Lammertink*[a]*

[a] Soft Matter, Fluidics and Interfaces, Faculty of Science and Technology, MESA+ Institute for Nanotechnology, University of Twente, Enschede, 7522 NB, The Netherlands

[b] School of Environment and Civil Engineering, Dongguan University of Technology, Dongguan, 523808, PR China

[c] TECNALIA, Basque Research and Technology Alliance (BRTA), Mikeletegi Pasealekua 2, 20009 Donostia, San Sebastian, Spain

***Author information*：**

Corresponding author:

Xiuqin Wang

E-mail: xiu-qin.wang@foxmail.com

Rob G. H. Lammertink

E-mail: R.G.H.Lammertink@utwente.nl

1. Computational Details

Computational and Simulation Details

The initial polymeric structures, both dry and wet were compressed and annealed into an amorphous periodic cuboidal cell by performing multi-step molecular dynamics simulations before the sampling of trajectories. First, the polymers were subjected to a high-pressure barostat at 1 GPa and a low timestep of 0.25 fs for 15,000 steps. This barostat step constricts the loose polymers into a compact cuboid. Once the initial cuboid is formed, the polymers undergo an annealing simulation procedure first proposed by Goddard and coworkers.[1-3]

This 3-step annealing procedure involves a series of expansion and compression processes:

a) Expansion of the system by 50% of initial volume by performing an MD simulation on an NPT ensemble at 1 atm pressure while increasing the temperature from 300 to 600 K for 50 ps.

b) 50 ps NVT MD simulations with the expanded volume at a temperature of 600 K.

c) Compression of the system back to initial volume by cooling it to 300 K for 50 ps by NPT MD at 1 atm pressure.

This 3-step procedure is repeated 5 times which is followed by a 100 fs NVT run at 300 K. After the annealing steps, the structures undergo a 5 ns equilibration run at the target temperature of 293.15 K. Once the systems are fully equilibrated, a production run is performed for 10 ns where the trajectories are sampled for calculating desired properties like diffusivities. The average box sizes of each system are as follows: dry linear polymer – $33.5 \times 25.6 \times 42.8$ Å^3, wet linear polymer – $25.9 \times 39.5 \times 47.9$ Å^3, dry crosslinked polymer – $32.4 \times 31.7 \times 33.8$ Å^3, wet crosslinked polymer – $37.0 \times 33.1 \times 38.7$ Å^3.

Force field

The systems have three different entities: polymer, counterions, and water. Therefore, three force fields were used for the simulations. The polymer is described using the AMBER force field, OH^- using the force field proposed by Han et al., and water using

Table S1. Summary of MD simulation runs.

Step	Substep	Ensemble	P (atm)	T (K)	Δt (fs)	N_{steps}	t_{MD} (ps)
1		Barostat	9869.23 (1 GPa)	300	0.25	15,000	3.75
	a	NPT	1	600	1	50,000	50
2	b	NVT	-	600	1	50,000	50
	c	NPT	1	300	1	50,000	50
	a	NPT	1	600	1	50,000	50
3	b	NVT	-	600	1	50,000	50
	c	NPT	1	300	1	50,000	50
4	a	NPT	1	600	1	50,000	50
	b	NVT	-	600	1	50,000	50

the SPC/E force field.[4,5] The form of the force field is as follows:

$$U_{total} = U_{bond} + U_{angle} + U_{dihedral} + U_{improper} + U_{coulomb} + U_{vdW}$$

where U_{total} – Total potential energy

U_{bond} – Bond energy in polymer and hydroxide

U_{angle} – Energy due to angles in polymer

$U_{dihedral}$ – Energy due to dihedrals in polymer

$U_{improper}$ – Energy due to improper dihedrals in polymer

$U_{coulomb}$ – Coulombic interaction energy between the charged atoms

U_{vdW} – Energy due to van der Waal's interactions between all atoms

The charges on polymer atoms were assumed to be concentrated on the nitrogen atom of QA, and therefore, the nitrogen atom was given a charge of +1. The van der Waals parameters were taken from the AMBER force field. The charges and van der Waal's parameters on hydroxyl ions and water are taken from Han et al. and the SPC/E force field, respectively.

Simulation Details

Details of the MD simulation runs performed for creating polymeric cells, equilibration, and production are given in Table S1.[1-3,5]

	c	NPT	1	300	1	50,000	50
5	a	NPT	1	600	1	50,000	50
	b	NVT	-	600	1	50,000	50
	c	NPT	1	300	1	50,000	50
6	a	NPT	1	600	1	50,000	50
	b	NVT	-	600	1	50,000	50
	c	NPT	1	300	1	50,000	50
7		NVT	-	300	1	100,000	100
8 (equilibration)		NPT	1	293.15 (target)	1	5,000,000	5,000
9 (production)		NPT	1	293.15	1	10,000,000	10,000

Simulation Results

Both static or structural and dynamic properties of the polymer systems were extracted from the trajectories produced during the 10 ns production atomistic MD run.

In general, the polymer system is divided into three atom centers – the polymer atom center (represented by the N atom), the hydroxide center (represented by the center of mass of the hydroxide ion, OH^-), and the water center (represented by water center of mass, OW).

The most probable displacement between each atom group can be calculated as radial distribution function (RDF), $g_{A-B}(r)$

$$g_{A-B}(r) = \frac{n_B}{4\Pi r^2 \Delta r} / (\frac{N_B}{V})$$

where $g_{A-B}(r)$ is the probability of finding two particles A and B at a distance of r, n_B is the number of particles B in the $(r, r + \Delta r)$ ring where $\Delta r = 0.1$ Å, N_B is the total number of particle B in the system and V is the total volume of the system. The coordination number of B around A can be calculated by integrating these curves.

Table S2. First coordination shell properties of each atom species in the systems.

**N-N**	**dry**		**wet**	
	m-TPNPiQA	C-FPVBC-1.7	m-TPNPiQA	C-FPVBC-1.7
r_{max}(Å)	6.3	6.7	8.9	8.7
$g_{A-B}(r_{max})$	3.5	2.4, 2.8	2.4	2.0
Coordination number	5.9	4.8	9.9	8.8
**N-OH**	**dry**		**wet**	
	m-TPNPiQA	C-FPVBC-1.7	m-TPNPiQA	C-FPVBC-1.7
r_{max}(Å)	4.5	3.7	4.9	4.9
$g_{A-B}(r_{max})$	11.7	13.0	2.9	2.4
Coordination number	9.2	9.8	10.66	9.4
**OH-OH**	**dry**		**wet**	
	m-TPNPiQA	C-FPVBC-1.7	m-TPNPiQA	C-FPVBC-1.7
r_{max}(Å)	6.1	6.5	4.2	4.2
$g_{A-B}(r_{max})$	3.1	2.8	9.2	10.4
Coordination number	5.3	6.6	4.9	5.5
**N-OW**	**dry**		**wet**	
	m-TPNPiQA	C-FPVBC-1.7	m-TPNPiQA	C-FPVBC-1.7
r_{max}(Å)	-	-	4.7	4.7
$g_{A-B}(r_{max})$	-	-	3.5	3.1
Coordination number	-	-	4.9	5.4
**OH-OW**	**dry**		**wet**	
	m-TPNPiQA	C-FPVBC-1.7	m-TPNPiQA	C-FPVBC-1.7
r_{max}(Å)	-	-	2.7	2.7

$g_{A-B}(r_{max})$	-	-	4.7	4.7
Coordination number	-	-	5.6	5.8
**OW-OW**	**dry**		**wet**	
	m-TPNPiQA	C-FPVBC-1.7	m-TPNPiQA	C-FPVBC-1.7
r_{max}(Å)	-	-	2.8	2.8
$g_{A-B}(r_{max})$	-	-	6.8	6.9
Coordination number	-	-	2.7	2.8

Table S3. Diffusivity values of polymer chain, hydroxide and water

Species	Diffusivity D_s ($* 10^{-7}$ cm^2/s)					
	Polymer chian		Hydroxide		Water	
	m-TPNPiQA	C-FPVBC-1.7	m-TPNPiQA	C-FPVBC-1.7	m-TPNPiQA	C-FPVBC-1.7
Dry	0.03	0.01	0.01	0.73	-	-
Wet	0.07	0.08	1.65	8.53	1.71	8.68

2. Experimental Data

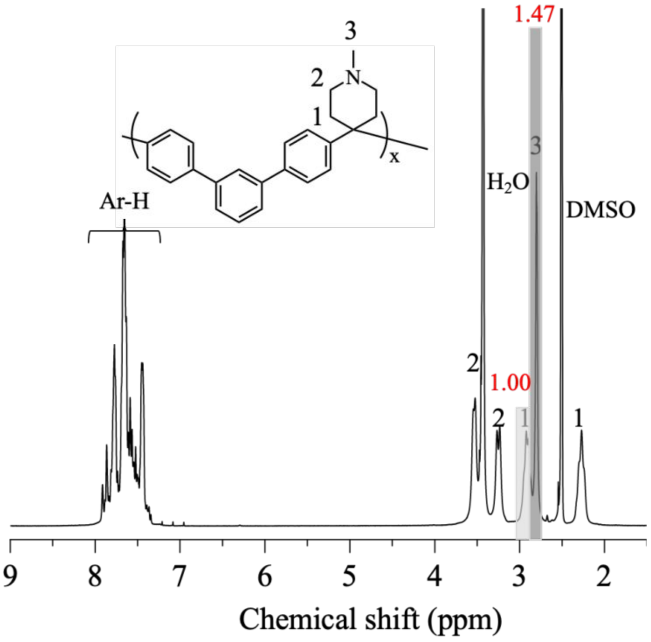

Figure S1. ^{1}H NMR spectrum of PAP.

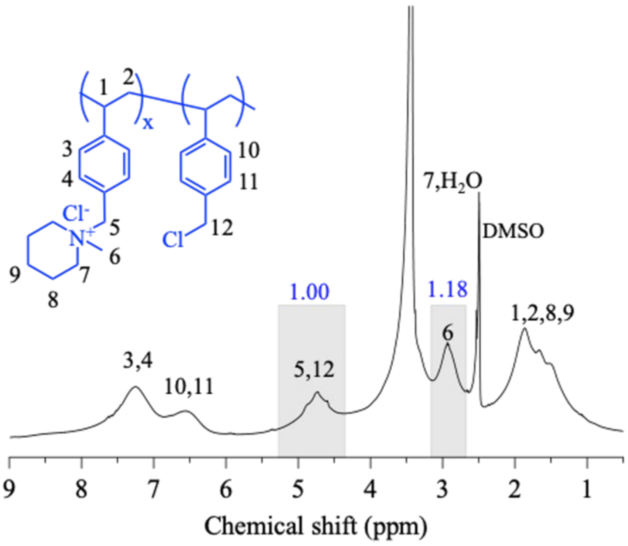

Figure S2. ^{1}H NMR spectrum of FPBVC.

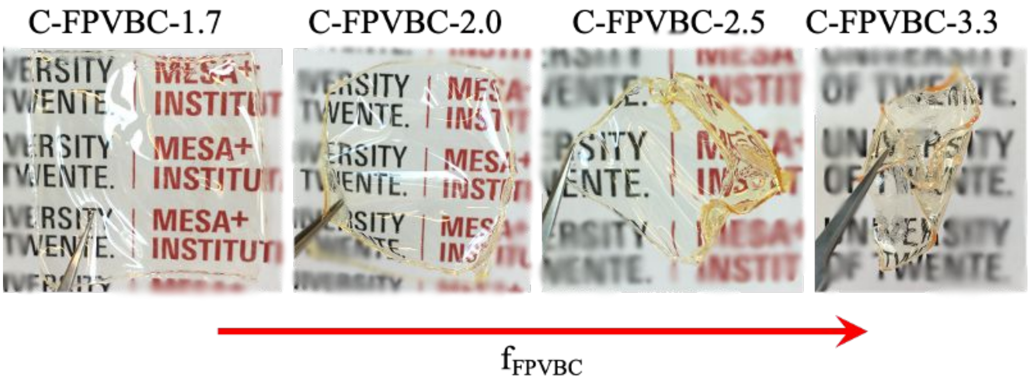

Figure S3. The appearance of crosslinked AEMs by increasing the ratio of FPVBC to PAP higher than 1.7.

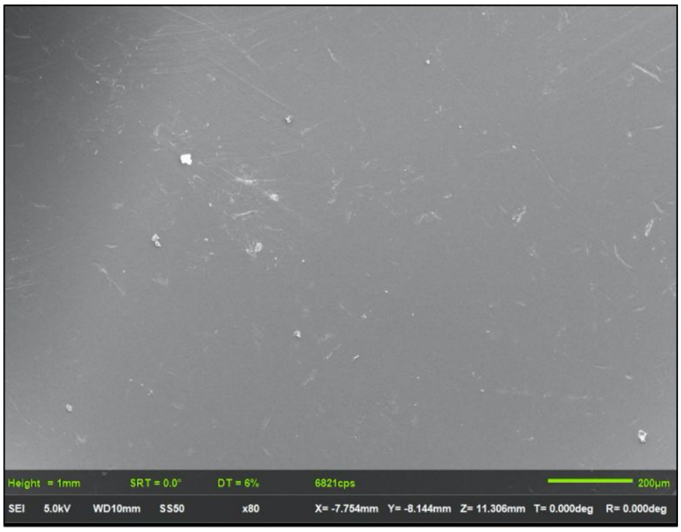

Figure S4. SEM image and membrane photo of C-FPVBC-1.7 AEM.

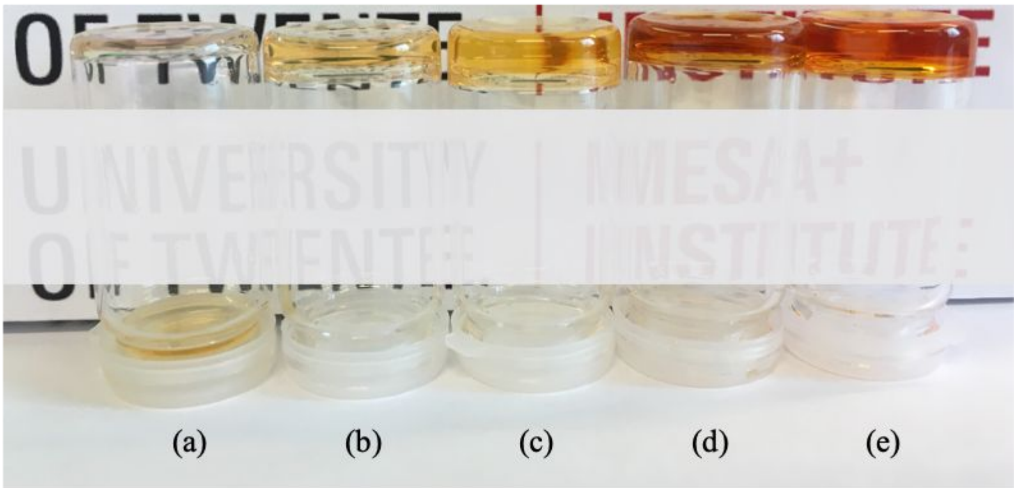

Figure S5. Crosslinking phenomenon during the preparation of the AEMs in various ratios at 80 °C for 48 h, (a) PAP and FPVBC solution, (b) C-FPVBC-0.7, (c) C-FPVBC-0.8, (d) C-FPVBC-1.2, and (e) C-FPVBC-1.7 solutions, respectively.

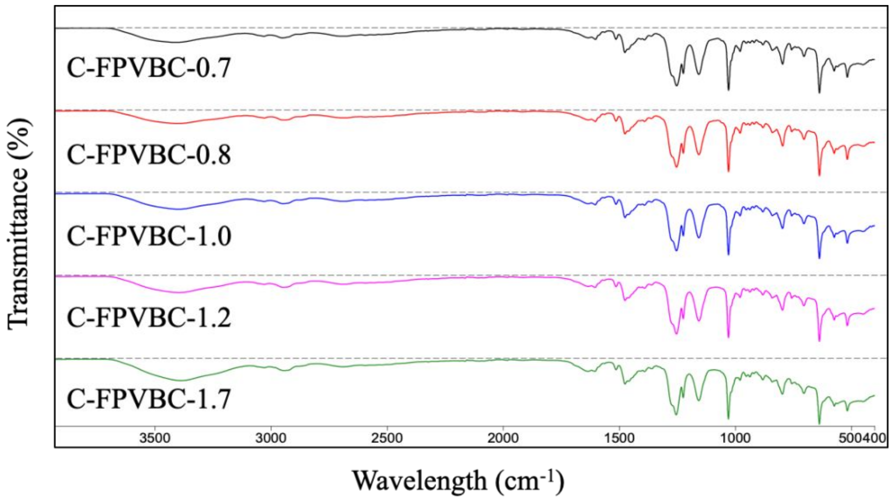

Figure S6. FT-IR spectra of serious C-FPVBC-x AEMs.

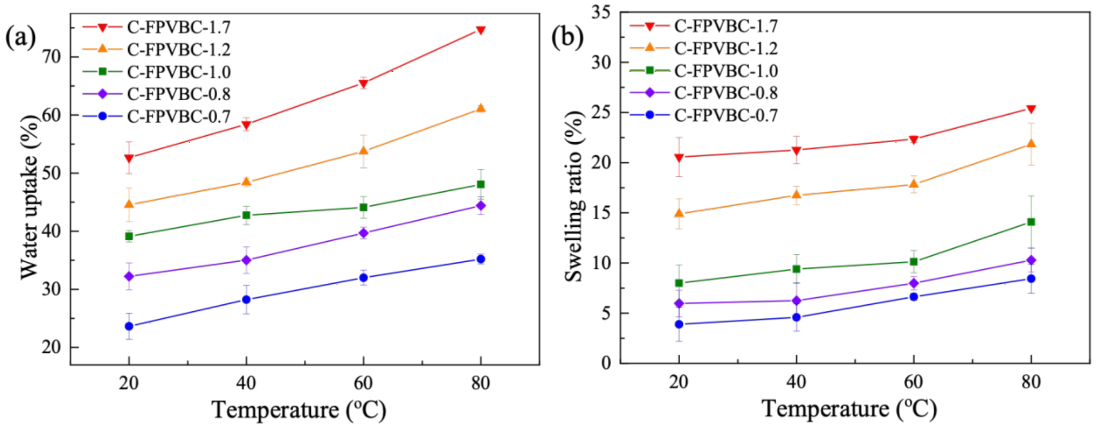

Figure S7. (a) WU and (b) SR of the C-FPVBC-x membranes as a function of temperature.

Table S4. Conductivity (σ), IEC, and σ/IEC of the AEMs.

AEMs	T(°C)	IEC (meq g^{-1})	σ (mS cm^{-1})	σ/IEC	Ref.
C-FPVBC-1.7	30	3.19	48.37	15.1	This work
C-FPVBC-1.7	80	3.19	77.15	24.2	This work
m-TPNPiQA	30	2.66	27.2	10.2	[6]
QMter-co-Mpi-80%	30	2.1	21.8	10.4	[7]
QMter-co-Mpi-100%	30	2.42	36.0	14.9	[8]
PBPIL1.55	30	1.55	25.9	16.7	[8]
PBPipQ47%-C35%	80	1.67	23	13.7	[9]
PBPipQ77%	80	2.83	56	19.8	[9]

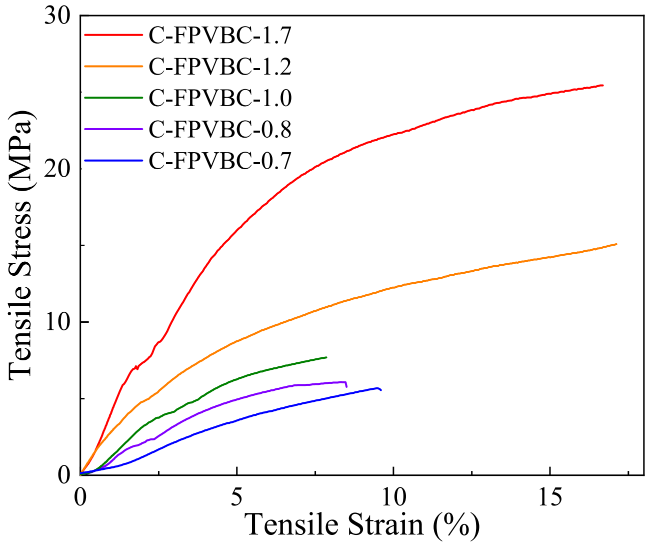

Figure S8. Strain–stress curves for C-FPVBC-x AEMs at room temperature.

Table S5. The mechanical properties of C-FPVBC-x AEMs

AEMs	Young's modulus (MPa)	Elongation at break (%)	Tensile strength (MPa)	Reference
C-FPVBC-0.7	88.63	9.66	5.67	This work
C-FPVBC-0.8	178.1	8.51	6.07	This work
C-FPVBC-1.0	196.38	7.86	7.69	This work
C-FPVBC-1.2	252.04	17.12	15.09	This work
C-FPVBC-1.7	478.28	16.69	25.45	This work
m-TPNPiQA	646.67	8.94	21.05	[10]
QMter-co-Mpi-100%	N/A	24	11	[11]
G-PPTPT-5:5	1330	6.7	37.7	[12]
Sustainion® 37-50	Cracks[a]	Cracks	Cracks	[13]
QAPVP-5%	N/A	3.5	7.4	[14]
c-AEM-23	2.03	4.48	37.1	[15]
90-10-QOH	262.76	44.35	22.27	[16]
PHF_xTP_{100-x}-PVBC	N/A	9.3	43.5	[17]

[a] in dry state.

Table S6. Comparison of cell durability performance with reported literature.

Membrane	Anode	Cathode	Electrolyte	Temperature	Performance	Durability
C-FPVBC-1.7	$NiFe_2O_4$	NiFeCo	1 M KOH	50 °C	2.4 V at 675 mA cm^{-2}	100 mA cm^{-2} for 100 h (2.0V)
QMter-co-Mpi-100%[18]	IrO_2	Pt/C	5.6wt% KOH	50 °C	2.0 V at 250 mA cm^{-2}	200 mA cm^{-2} for 500 h (2.1V)

PSEBS-CM-DABCO[19]	$NiCo_2O_4$	$NiFe_2O_4$	15wt% KOH	50 °C	2.0 V at 150 mA cm^{-2} (40 °C)	300 mA cm^{-2} for 150 h (2.267 V)
PPO24-BIM[20]	IrO_2	Pt/C	0.5 M KOH	50 °C	1.8 V at 318 mA cm^{-2}	N/A
SEBS-Pi[21]	IrO_2	Pt/C	1 M KOH	50 °C	2.0 V at 400 mA cm^{-2}	400 mA cm^{-2} for 105 h (2.08 V)
C-ABPBI[22]	Ni foam	Ni foam	16.8wt% KOH	70 °C	2.0 V at 335 mA cm^{-2}	N/A
PAEK-APMBI[23]	Ni foam	Ni foam	10wt% KOH	60 °C	1.9 V at 500 mA cm^{-2}	N/A

Table S7. The R_m, R_c, and R_a of electrolyzer based on C-FPVBC-1.7.

Temperature (°C) at 1.8 V	R_m (Ω cm^2)	R_c (Ω cm^2)	R_a (Ω cm^2)
RT	0.92	2.05	8.84
50	0.90	1.00	3.39
80	0.84	0.34	2.74
Voltage (V) at 50 °C	R_m (Ω cm^2)	R_c (Ω cm^2)	R_a (Ω cm^2)
1.5	0.92	1.14	-
1.8	0.90	1.00	13.13
2.1	0.91	0.30	7.10

Reference

(1) Jang, S. S.; Molinero, V.; Çağın, T.; Goddard, W. A., Nanophase-Segregation and Transport in Nafion 117 from Molecular Dynamics Simulations: Effect Of Monomeric Sequence. *J. Phys. Chem. B* **2004**, 108, 3149–3157.

(2) Jang, S. S.; Lin, S. T.; Çağın, T.; Molinero, V.; Goddard, W. A., Nanophase Segregation and Water Dynamics in the Dendrion Diblock Copolymer Formed from the FrÉchet Polyaryl Ethereal Dendrimer and Linear PTFE. *J. Phys. Chem. B* **2014**, 118, 12577–12587.

(3) Chang, H.; Wang, P.; Li, H.; Zhang, J.; Yan, D., Solvent Vapor Assisted Spin-Coating: A Simple Method to Directly Achieve High Mobility from P3HT Based Thin Film Transistors. *Synth. Met.* **2013**, 184, 1–4.

(4) Abascal, J. L.; Vega, C., A Systematic Study of Water Models for Molecular Simulation: Derivation of Water Models Optimized for Use with A Reaction Field. *J. Chem. Phys.* **2005**, 123, 234505.

(5) Han, K. W.; Ko, K. H.; Abu-Hakmeh, K.; Bae, C.; Sohn, Y. J.; Jang, S. S., Molecular Dynamics Simulation Study of a Polysulfone-Based Anion Exchange Membrane in Comparison with the Proton Exchange Membrane. *J. Phys. Chem. C* **2014**, 118, 12577–12587.

(6) Wang, X.; Lin, C.; Gao, Y.; Lammertink, R. G. H., Anion Exchange Membranes with Twisted Poly(terphenylene) Backbone: Effect of the N-cyclic Cations. *J. Membr. Sci.* **2021**, 635, 119525.

(7) Yan, X.; Yang, X.; Su, X.; Gao, L.; Zhao, J.; Hu, L.; Di, M.; Li, T.; Ruan, X.; He, G., Twisted Ether-free Polymer Based Alkaline Membrane for High-Performance Water Electrolysis. *J. Power Sources* **2020**, 480, 228805.

(8) Ma, L.; Hussain, M.; Li, L.; Qaisrani, N. A.; Bai, L.; Jia, Y.; Yan, X.; Zhang, F.; He, G., Octopus-like Side Chain Grafted Poly(arylene piperidinium) Membranes for Fuel Cell Application. *J. Membr. Sci.* **2021**, 636, 119529.

(9) Olsson, J. S.; Pham, T. H.; Jannasch, P., Tuning Poly(arylene piperidinium) Anion-Exchange Membranes by Copolymerization, Partial Quaternization and Crosslinking. *J. Membr. Sci.* **2019**, 578, 183–195.

(10) Wang, X.; Lammertink, R. G. H., Dimensionally Stable Multication-Crosslinked Poly(arylene piperidinium) Membranes for Water Electrolysis. *J. Mater. Chem. A* **2022,** *10*, 8401–8412.

(11) Yan, X.; Yang, X.; Su, X.; Gao, L.; Zhao, J.; Hu, L.; Di, M.; Li, T.; Ruan, X.; He, G., Twisted Ether-Free Polymer Based Alkaline Membrane for High-Performance Water Electrolysis. *J. Power Sources* **2020,** *480*, 228805.

(12) Wang, Q.; Huang, L.; Zheng, J.; Zhang, Q.; Qin, G.; Li, S.; Zhang, S., Design,

Synthesis and Characterization of Anion Exchange Membranes Containing Guanidinium Salts with Ultrahigh Dimensional Stability. *J. Membr. Sci.* **2022,** *643*, 120008.

(13) Henkensmeier, D.; Najibah, M.; Harms, C.; Žitka, J.; HnÁt, J.; Bouzek, K., Overview: State of the Art Commercial Membranes for Anion Exchange Membrane Water Electrolysis. *J. Electrochem. Energy Convers. Stor.* **2021,** *18* (2), 024001–18.

(14) Li, L.; Yang, Q.; Gao, X. L.; Wu, H. Y.; Zhang, Q. G.; Zhu, A. M.; Liu, Q. L., Facile Construction of Crosslinked All-Carbon-Backbone Anion-Exchange Membranes with Robust Durability. *J. Materi. Chem. A* **2018,** *6*, 24831–24840.

(15) Xue, J.; Liu, L.; Liao, J.; Shen, Y.; Li, N., UV-Crosslinking of Polystyrene Anion Exchange Membranes by Azidated Macromolecular Crosslinker for Alkaline Fuel Cells. *J. Membr. Sci.* **2017,** *535*, 322–330.

(16) Arslan, F.; Chuluunbandi, K.; Freiberg, A. T. S.; Kormanyos, A.; Sit, F.; Cherevko, S.; Kerres, J.; Thiele, S.; Böhm, T., Performance of Quaternized Polybenzimidazole-Cross-Linked Poly(vinylbenzyl chloride) Membranes in HT-PEMFCs. *ACS Appl. Mater. Interfaces* **2021**, *13* (47), 56584–56596.

(17) Xu, F.; Chen, Y.; Li, J.; Lin, B.; Chu, F.; Ding, J., Polyfluorene/Poly(vinylbenzyl chloride) Cross-Linked Anion-Exchange Membranes with Multiple Cations for Fuel Cell Applications. *ACS Appl. Energy Mater.* **2022**, *5* (7), 9101–9108.

(18) Yan, X.; Yang, X.; Su, X.; Gao, L.; Zhao, J.; Hu, L.; Di, M.; Li, T.; Ruan, X.; He, G., Twisted Ether-Free Polymer Based Alkaline Membrane for High-Performance Water Electrolysis. *J. Power Sources* **2020**, 480, 228805.

(19) Hnát, J.; Plevová, M.; Žitka, J.; Paidar, M.; Bouzek, K., Anion-Selective Materials with 1,4-Diazabicyclo[2.2.2]octane FunctionalGroups for Advanced Alkaline Water Electrolysis. *Electrochim. Acta* **2017**, 248, 547–555.

(20) Marinkas, A.; Strużyńska-Piron, I.; Lee, Y.; Lim, A.; Park, H. S.; Jang, J. H.; Kim, H. J.; Kim, J.; Maljusch, A.; Conradi, O.; Henkensmeier, D., Anion-Conductive Membranes Based on 2-Mesityl-Benzimidazolium Functionalized Poly(2,6-dimethyl-

1,4-phenylene oxide) and Their Use in Alkaline Water Electrolysis. *Polymer* **2018**, 145, 242–251.

(21) Su, X.; Gao, L.; Hu, L.; Qaisrani, N. A.; Yan, X.; Zhang, W.; Jiang, X.; Ruan, X.; He, G., Novel Piperidinium Functionalized Anionic Membrane for Alkaline Polymer Electrolysis with Excellent Electrochemical Properties. *J. Membr. Sci.* **2019**, 581, 283–292.

(22) Diaz, L. A.; Hnát, J.; Heredia, N.; Bruno, M. M.; Viva, F. A.; Paidar, M.; Corti, H. R.; Bouzek, K.; Abuin, G. C., Alkali Doped Poly(2,5-benzimidazole) Membrane for Alkaline Water Electrolysis: Characterization and Performance. *J. Power Sources* **2016**, 312, 128–136.

(23) Tham, D. D.; Kim, D., C_2 and N_3 Substituted Imidazolium Functionalized Poly(arylene ether ketone) Anion Exchange Membrane for Water Electrolysis with Improved Chemical Stability. *J. Membr. Sci.* **2019**, 581, 139–149.
